# Development of a microphysiological skin-liver-thyroid Chip3 model and its application to evaluate the effects on thyroid hormones of topically applied cosmetic ingredients under consumer-relevant conditions

**DOI:** 10.3389/fphar.2023.1076254

**Published:** 2023-02-08

**Authors:** Thi-Phuong Tao, Ilka Maschmeyer, Edward L. LeCluyse, Eda Rogers, Katrin Brandmair, Silke Gerlach, Julia Przibilla, Fredy Kern, Camille Genies, Carine Jacques, Abdulkarim Najjar, Andreas Schepky, Uwe Marx, Jochen Kühnl, Nicola J. Hewitt

**Affiliations:** ^1^ TissUse GmbH, Berlin, Germany; ^2^ LifeNet Health, Durham, NC, United States; ^3^ LifeNet Health, Virginia Beach, VA, United States; ^4^ Beiersdorf AG, Hamburg, Germany; ^5^ Pharmacelsus GmbH, Saarbrücken, Germany; ^6^ Pierre Fabre Dermo-Cosmétique, Toulouse, France; ^7^ Cosmetics Europe, Auderghem, Belgium

**Keywords:** microphysiological systems, cosmetics, HUMIMIC Chip3, thyroid follicles, endocrine disruption, case study, genistein, daidzein

## Abstract

All cosmetic ingredients registered in Europe must be evaluated for their safety using non-animal methods. Microphysiological systems (MPS) offer a more complex higher tier model to evaluate chemicals. Having established a skin and liver HUMIMIC Chip2 model demonstrating how dosing scenarios impact the kinetics of chemicals, we investigated whether thyroid follicles could be incorporated to evaluate the potential of topically applied chemicals to cause endocrine disruption. This combination of models in the HUMIMIC Chip3 is new; therefore, we describe here how it was optimized using two chemicals known to inhibit thyroid production, daidzein and genistein. The MPS was comprised of Phenion^®^ Full Thickness skin, liver spheroids and thyroid follicles co-cultured in the TissUse HUMIMIC Chip3. Endocrine disruption effects were determined according to changes in thyroid hormones, thyroxine (T_4_) and 3,3’,5-triiodothyronine (T_3_). A main part of the Chip3 model optimization was the replacement of freshly isolated thyroid follicles with thyrocyte-derived follicles. These were used in static incubations to demonstrate the inhibition of T_4_ and T_3_ production by genistein and daidzein over 4 days. Daidzein exhibited a lower inhibitory activity than genistein and both inhibitory activities were decreased after a 24 h preincubation with liver spheroids, indicating metabolism was *via* detoxification pathways. The skin-liver-thyroid Chip3 model was used to determine a consumer-relevant exposure to daidzein present in a body lotion based on thyroid effects. A “safe dose” of 0.235 μg/cm^2^ i.e., 0.047% applied in 0.5 mg/cm^2^ of body lotion was the highest concentration of daidzein which does not result in changes in T_3_ and T_4_ levels. This concentration correlated well with the value considered safe by regulators. In conclusion, the Chip3 model enabled the incorporation of the relevant exposure route (dermal), metabolism in the skin and liver, and the bioactivity endpoint (assessment of hormonal balance i.e., thyroid effects) into a single model. These conditions are closer to those *in vivo* than 2D cell/tissue assays lacking metabolic function. Importantly, it also allowed the assessment of repeated doses of chemical and a direct comparison of systemic and tissue concentrations with toxicodynamic effects over time, which is more realistic and relevant for safety assessment.

## 1 Introduction

All cosmetic ingredients must be evaluated for their safety prior to their use in products. As the use of animals for such assessments in Europe has been completely banned since 2013, cosmetic ingredients are now being evaluated using non-animal methods employed in a tiered approach (Desprez et al., 2018; Alexander-White et al., 2022). Microphysiological systems (MPS) incorporating cellular models representing different organs offer a more complex higher tier model that could be used to evaluate effects of test chemicals (Thomas et al., 2019). Moreover, since the route of exposure to most cosmetic ingredients is dermal, MPS also offer the opportunity to incorporate skin models to mimic topical exposure and provide consumer-relevant data regarding leave-on/rinse-off products. For example, a skin and liver HUMIMIC Chip2 co-culture model was used to demonstrate how the dosing frequency and application route impacted the kinetics of test chemicals and their metabolites (Kühnl et al., 2021; Tao et al., 2021). Importantly, the skin-liver Chip2 model also recapitulated the first-pass effect of the skin and alterations in the metabolite profile of a hair dye 4-amino-2-hydroxytoluene observed *in vivo* (Tao et al., 2021).

A highly debated concern for some cosmetics ingredients is their potential to cause endocrine disruption and subsequent impairment of reproduction (Nicolopoulou-Stamati et al., 2015; Gore and Cohn, 2020; Rubin and Brod, 2020). It is important to consider not just the hazard potential of these chemicals but also the exposure due to cosmetics use, which may be insignificant compared to other routes and sources of the same chemical (Rubin and Brod, 2020). Therefore, we investigated whether the skin-liver Chip2 co-culture model could be adapted to incorporate a third tissue model (referred to here as an “organoid”) to evaluate the potential of chemicals to cause endocrine disruption under consumer-relevant conditions. While there are currently no suitable tissue constructs for MPS models representing endocrine disruption due to estrogenic effects, a HUMIMIC liver-thyroid Chip2 model has been established to investigate both direct and indirect thyroid disruptors (Kühnlenz et al., 2022). This model used freshly isolated human thyroid follicles in a co-culture with liver spheroids (Kühnlenz et al., 2022). The interconnection between the thyroid and the liver is important since the liver plays a role in thyroid hormone distribution, metabolism and elimination and supplies thyroglobulin required for the storage of thyroid hormones (Boerescu, 1991).

**FIGURE 1:**
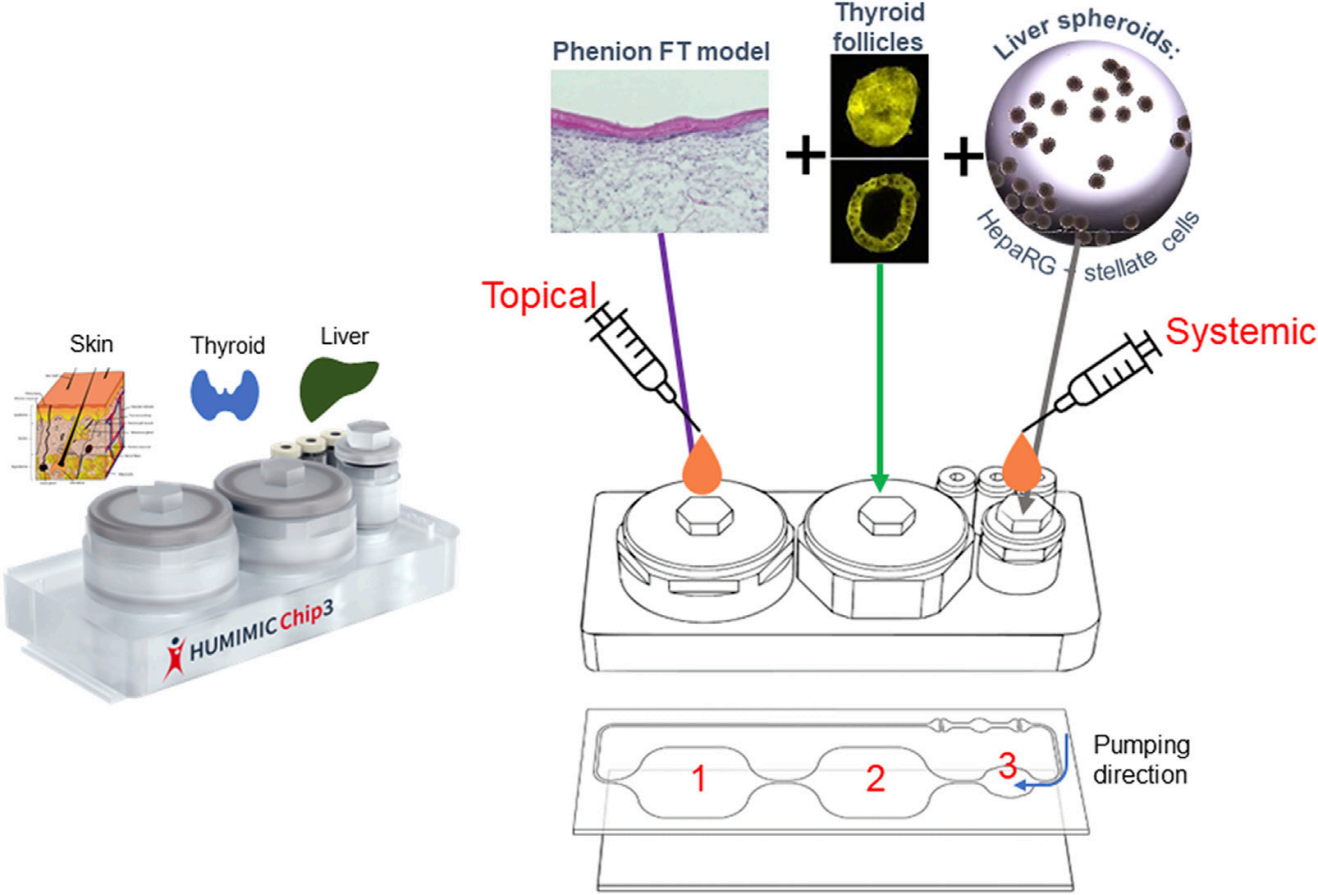
Set up of the skin-liver-thyroid Chip3 model.

The combination of the skin, liver and thyroid in the HUMIMIC liver-thyroid Chip3 has so far not been developed; therefore, we describe here how this new Chip3 model was optimized using two chemicals known to cause endocrine disruption by inhibiting thyroid production, namely daidzein and genistein (Scientific Committee on Consumer Safety, 2022). Bioassay data from the US Environmental Protection Agency ToxCast Screening Library indicate that the biological activity of daidzein is at least an order of magnitude lower than genistein (United States Environmental Protection Agency, 2007). Potential endocrine disruption effects of the test chemicals were analyzed according to changes in the production of the major thyroid hormones, thyroxine (T_4_) and 3,3’,5-triiodothyronine (T_3_). The resulting model (shown in Figure 1) enables the incorporation of the relevant exposure route of the chemicals (dermal), metabolism in the skin and liver, and the bioactivity endpoint (thyroid effects) in a single model. Importantly, it also allows the assessment of repeated doses of test chemicals and a direct comparison of systemic and tissue concentrations with toxicodynamic effects over time.

A technical issue faced in this work was to develop a reliable protocol to generate functional thyroid follicle-like organoids from cryopreserved thyrocytes, as the access to fresh human thyroid tissue for the isolation of thyroid follicles was a limiting factor. We describe the steps taken to generate, maintain and qualify the thyrocyte-derived thyroid follicles, and the adaptation of the conditions in the Chip3 model to maintain all three tissues in co-culture. The qualification of the thyrocyte-derived thyroid follicles included testing a known inhibitor of thyroid hormone production, namely methimazole (MMI), which inhibits thyroid peroxidase (a key enzyme involved in thyroid hormone synthesis) (Paul et al., 2014).

Finally, we demonstrate in a hypothetical consumer-relevant case study how the skin-liver-thyroid Chip3 model could be used to determine the highest concentrations of genistein and daidzein in a body lotion which does not affect thyroid hormones.

## 2 Materials and methods

### 2.1 Materials

Daidzein (product code D7802) was from Sigma Aldrich and genistein (product number AG-CN2-0427) was from AdipoGen. Methimazole (MMI) product number 301507 was from Sigma Aldrich. All chemicals were of the highest purity. Bovine pituitary Thyroid Stimulating Hormone (TSH) was from Millipore (product number 609385-5IU). Charcoal-stripped fetal calf serum (FCS) was from either Sigma (product number F6765) or from Gibco (product number A33821-01). A detailed list of cells and media components is shown in Supplementary Material S1.

The body lotion formulation is a proprietary base formulation from Beiersdorf AG. The final preparations of genistein and daidzein were prepared at Pierre Fabre, Toulouse, France.

### 2.2 Skin and liver organoids

Liver spheroids were generated as described by Tao et al. (2021) using cryopreserved human stellate cells (HHSteC, product number 5300) from Provitro (Berlin, Germany) and differentiated HepaRG cells (HPR116, Lot HPR116239-TA08) from Biopredic International (Rennes, France). Each liver organoid contained 1,000 HHSteC and 24,000 HepaRG cells. Phenion^®^ full thickness (FT) skin models (FT INSERT24-1) and corresponding “air-liquid interface” (ALI) medium (ALI-CM-250) were from Phenion, Düsseldorf, Germany.

### 2.3 Isolation and culture of intact thyroid follicles and thyrocyte-derived thyroid follicles

An overview of the thyroid culture methods and designs of the static and Chip3 experiments are shown in Supplementary Figures S1, S2, respectively. Intact thyroid follicles were isolated from fresh human thyroid tissue according to Kühnlenz et al. (2022). Fresh human thyroid tissue (Lot 13202007TG112) was ethically obtained with full donor consent from Provio, Berlin, Germany. Follicles (500 or 1,000) were entrapped in 50 µL Matrigel and maintained in 0.5 mL medium over 10 days, with a 50% medium exchange every 2–3 days. The media tested were 100% liver-thyroid co-culture medium from Kühnlenz et al. (2022) and a mix of liver-thyroid co-culture medium and skin ALI medium (in a ratio of 50:50 or 70:30), all with or without the addition of 1 mU/mL TSH. The co-culture medium was phenol red-free SILAC Advanced DMEM/F-12 Flex Medium (Thermo Fisher, Germany) supplemented with 2 mM L-glutamine, 0.1 μM sodium iodine, 2 g/L glucose, 0.1 μM dexamethasone, 1% 50×MEM amino acids, 250 ng/mL amphotericin, 5 μg/mL gentamycin, 0.5% BSA and 10% charcoal-stripped FCS.

For all further experiments, cryopreserved human thyrocytes were used. These were either from LifeNet Health (Research Triangle Park, NC, United States) (Donors 1 (batch THY2016499), 3 (batch THY2016525), 4 (batch THY2014322), and 5 (batch THY2021448) or TissUse (Donors 2 (batch TU61) and 6 (batch TU60). The h7H medium was Nutrient Mixture F12 Ham Coons modification with L-glutamine and zinc sulfate, supplemented with 10% charcoal-stripped FCS and a “full supplement mix”: 2.68 g/L NaHCO_3_, 1% penicillin/streptomycin, 0.0025 μg/mL amphotericin B, 25 mU/mL insulin, 0.011 μg/L hydrocortisone, 0.2 μg/L human growth hormone, 5 mg/L transferrin, 0.067 µM NaI, 0.075 mg/L sodium selenite, 0.2 mg/mL reduced L-glutathione and 0.5 mg/L tocopherol.

Method 1 involved mixing 150,000 cells in 50 µL Matrigel in a 1.5 mL reaction tube and allowing them to gel for 24 h before being transferred to 48-well plates or the Chip3 circuit. For adaptation to the Chip3 circuit, two additional thyroid follicle methods were tested using thyrocytes from three donors (Donors 1, 2 and 3). In Method 2, cells were thawed and cultured on top of Matrigel in 96-well round-bottom plates at 15,000 cells per well in 0.1 mL h7H medium and 1 mU/mL TSH, according to Deisenroth et al. (Deisenroth et al., 2020). Method 3 involved seeding 15,000 cells in 0.1 mL h7H medium and 1 mU/mL TSH on top of Matrigel in 96-well round-bottom plates, and after 4 days the follicles were overlayed with 0.1 mL Matrigel, creating a thyroid follicle-containing bead. After 24 h, gels were transferred to 48-well plates or the Chip3 circuit. For all culture formats, the medium was changed every 2–3 days.

### 2.4 Static incubations—Optimization of thyrocyte cultures

In parallel with the first pilot Chip3 experiment, thyrocyte-derived thyroid follicles from the same donor (Donor 1) were also incubated in static cultures according to Method 2. Static cultures were incubated in 0.1 mL 100% h7H medium and 1 mU/mL TSH until Day 6 and with or without TSH from Day 6 until Day 14. A single application of 10 µM MMI was added to the medium of cultures on Day 10.

To optimize the culture method for thyroid follicles used in the Chip3 circuits, static incubations of thyrocyte-derived thyroid follicles (Donor 1, 2 and 3) were generated according to Methods 1, 2, and 3 using a modified medium considered to be suitable for the Chip3 incubations: 50% ALI medium and 50% h7H medium containing 10% FCS or 0.5% BSA. Cells were preincubated for 7 days in 1 mU/mL TSH, after which, the medium was changed every 2–3 days and replaced with fresh medium with or without 1 mU/mL TSH. All medium removed from the cultures over 14 days was analyzed for the presence of T_3_ and T_4_. All treatments were run in triplicate.

### 2.5 Static incubations—Genistein and daidzein concentration-response curves

To evaluate the inhibitory potential of genistein and daidzein in thyroid follicles produced using the optimized culture method (Method 3), thyroid follicles from two donors (Donor 1 and 4) were transferred to 48-well plates (0.5 mL, two follicle-containing beads/well and one bead per donor). During the formation of follicles, half the medium was replaced on Day 1 and 4. The medium was 50% ALI medium and 50% h7H medium containing the full supplement mix, 0.5% BSA and 1 mU/mL TSH. On Day 7, half the medium was removed and replaced with fresh medium containing 2x the final concentration of test chemicals (0.1–30 µM daidzein or 0.1–30 µM genistein in ethanol), the solvent control (the final concentration in the thyroid follicles was 0.15%–0.5% ethanol or 1% DMSO) or positive control (10 µM MMI in DMSO). Cells were treated daily for 4 days using the half medium exchange with fresh medium.

In parallel, all test chemicals and solvent controls were preincubated with liver organoids for 24 h prior to adding to the thyroid follicles. To enable the reproducible application of identically-treated thyroid follicles in triplicate over 4 days, these pre-incubations were staggered - each test chemical and solvent were incubated with 20 spheroids/well in 0.8 mL medium, each incubation starting 24 h before transfer to the thyroid follicles. After 24 h, 3 × 0.25 mL medium was removed from one treatment well and added to three wells containing 0.25 mL medium with two thyroid follicles (in a 48-well plate). The process was repeated four times—one for each day. The same medium was used for liver and thyroid organoid incubations.

The media from the liver organoid preincubations were analyzed for the presence of test chemicals and metabolites and the media from the thyroid follicle incubations were analyzed for T_3_ and T_4_. All treatments were run in triplicate. The concentrations of genistein and daidzein remaining after each 24 h incubation are shown in the Supplementary Material S2.

To calculate the IC_50_ values for incubations conducted on each day, the T_4_ and T_3_ concentrations were plotted against the nominal concentration transformed to log10. The curves were then fitted to a log (agonist) vs. response (3 parameter) best fit using Prism GraphPad software. The table of results in the software provides the goodness of fit, as well as the IC_50_ value.

### 2.6 Chip3 experiments

#### 2.6.1 Basic Chip3 setup and medium sampling

The Chip3 circuits were filled with medium at 1 day prior to adding the organoids in the chip. On the day of the Chip3 assembly, the organoids were transferred to the compartments of the Chip3: 40 liver spheroids in one compartment, one Phenion FT skin model in a Millicell culture insert was integrated into a second culture compartment and two to four Matrigel beads containing thyroid follicles were transferred to the third compartment. The circuit was filled with 1 mL fresh medium. The Chip3 circuits were connected to HUMIMIC Starter, the pump control units, operating at a pressure of 350 mbar and vacuum of 300 mbar with 0.5 Hz as the pump frequency. The chips were only removed from the Starter during medium exchange, in which 0.5 mL medium was removed and replaced with the same volume of appropriate fresh medium.

#### 2.6.2 Chip3 pilot

In the first pilot Chip3 experiment using thyrocyte-derived thyroid follicles, the follicles were generated using Method 1 (and thyrocytes from Donor 1) and precultured in static culture for 8 days before being transferred to the Chip3. The Chip3 medium was a ratio of 50:50 ALI medium to h7H medium containing 0.5% BSA, with and without 1 mU/mL TSH. Four days after the Chip3 organoid assembly, circuits were treated with a single dose of 10 µM MMI (Day 12, final circuit concentration) or solvent control (0.1% DMSO). The medium was fully replaced on Day 14 without MMI and the incubation continued until Day 16. All media from the static incubations and Chip3 medium changes were analyzed for T_3_ and T_4_. All treatments were run in five replicate chips.

#### 2.6.3 Chip3—Dose selection for genistein and daidzein

The aim of the final Chip3 experiments was to measure T_4_ and T_3_ levels in the Chip3 after topical doses of daidzein and genistein and compare these with T_4_ and T_3_ levels after systemic doses of daidzein and genistein which were expected to alter T_4_ and T_3_ levels. Systemic and topical doses of genistein and daidzein were based on their lowest observed effect (plasma) concentrations (LOEC), estimated from the preliminary static incubations with thyroid follicles described above and results from the thyroid peroxidase inhibition assay (See Supplementary Material S3). The LOECs from the thyroid peroxidase inhibition assay were 19 ± 8.9 µM for genistein and 28 ± 13 µM for daidzein. To ensure systemic doses approximating the LOECs in the Chip3, the metabolism of the test chemicals by liver organoids (i.e., first-pass metabolism in the Chip) was taken into account. This was achieved by adjusting the nominal doses according to the correlation between the nominal concentration added and the concentration remaining after 24 h incubation with liver spheroids (see Supplementary Material S2 for the detailed calculation). The adjustment factors for genistein and daidzein were 1.39 and 2.1, respectively, resulting in doses of 25 µM genistein and 58 µM daidzein being applied.

Following the rationale of applying safety assessment factors to derive safe concentrations in conventional cosmetic safety assessment (Yang et al., 2017), the LOECs were divided by three to obtain a “no observed effect concentration” (NOEC). The NOECs were divided by 100 as a Margin of Safety (MoS) typically considered safe by regulatory authorities. After application of the safety factors, the corresponding “safe” steady state plasma concentrations were estimated to be 63 and 93 nM for genistein and daidzein, respectively. Subsequently, reverse physiologically based pharmacokinetic (PBPK) modelling was applied to calculate a topical dose for each chemical that would translate to the respective safe plasma concentration in the human population i.e., 63 and 93 nM for genistein and daidzein, respectively. The main parameters for the simulation were chosen by combining parameters for a body lotion exposure scenario (60 kg body weight, 15,760 cm^2^ body surface recommended by the Scientific Committee on Consumer Safety (SCCS) (Scientific Committee on Consumer Safety, 2021)) and experimental results for bioavailability: 76% for daidzein and 91% for genistein (mean+1SD, based on skin absorption in native frozen human skin and Phenion FT models (data not shown)). The parameters in the SCCS notes of guidance were slightly modified to meet experimental requirements: Instead of 2.28 applications per day (the mean frequency of body lotion use), we applied a single dose per day and, instead of the recommended application of 0.5 mg/cm^2^ for body lotions, we applied 5 mg/cm^2^ as this amount resulted in an even spread of lotion across the entire skin model. Based on these factors, doses for the first Chip3 experiment were determined to be 1.55 and 2.35 μg/cm^2^ for genistein and daidzein, respectively, and were expected to be “no observed effect level” (NOEL) doses. Since this was not the case (see Section 3.4.1), a second experiment was performed. The topical doses of daidzein for the second experiment were 2.35 μg/cm^2^ and a 10-fold lower dose of 0.235 μg/cm^2^ to derive a “safe dose” that would not be expected to alter hormone levels. The standard conversion of 3-fold from a LOEL to a NOEL, according to Yang et al. (2017) was considered to have insufficient confidence with respect to the resulting hormone inhibition; therefore, the overall safety factor was increased to 10.

#### 2.6.4 Chip3—Effect of genistein and daidzein on TSH stimulated T_4_ and T_3_

The medium used for the final two Chip3 experiments was a ratio of 30:70 ALI to h7H medium, containing the full supplement mix, 0.5% BSA and either 1 mU/mL TSH or 0.1 mU/mL TSH. One day after inoculation of the Chip3 with skin, liver and thyroid organoids (Day 6), the exposure to test chemicals, solvent control or MMI was started. For systemic application of the test chemicals, genistein and daidzein (both dissolved in 100% ethanol in the first experiment and in 100% DMSO in the second experiment) or MMI (dissolved in 100% DMSO in both experiments), the working solutions (or solvent controls) were mixed with 500 µL medium, which was then added to the liver compartment after removal of 500 µL medium. For genistein and daidzein in the first experiment, the final concentration of ethanol in the Chip3 was 2% (v/v) and in the second experiment, the final concentration of DMSO in the Chip3 was 0.1% (v/v). For MMI, the final concentration of DMSO in the Chip3 was 0.1% (v/v) in both experiments. For topical application, 3 mg of the lotion formulation containing the nominal doses of daidzein or genistein was applied to the top of the Phenion FT model (5 mg/cm^2^ to 0.6 cm^2^ surface area). The test chemicals, solvent controls and MMI were applied daily for 4 or 5 days. Treatments were run in 3–5 chips. After every 24 h, a volume of 0.5 mL medium was removed from the circuits and replaced with 0.5 mL of fresh medium (with or without test chemical depending on the treatment scenario).

For the analysis of test chemicals and their metabolites and T_3_ and T_4_, a volume of 80 µL of the sampled medium was aliquoted into 1.5 mL tubes or 96-well plates and stored at −80°C until analysis by LC-MS/MS. For metabolite analysis only, samples of medium were removed after 4 h (from separate circuits per timepoint such that only 80 µL was removed from each). All remaining supernatants were transferred into 96-deep well plates and stored at 4°C until lactate dehydrogenase (LDH), glucose, lactate and albumin analyses were performed. Tissues were harvested at the end of the incubation and processed further for histochemical analysis or analysis by LC-MS/MS.

### 2.7 Organoid structure and viability measurements

Albumin content of the medium was measured using Albumin in Urine/CSF FS Kit from DiaSys (Holzheim, Germany). Lactate concentrations in the medium were measured using the FluitestR Lactate kit from Analyticon (Lichtenfels, Germany). Glucose concentrations in the medium were measured using the Glucose (HK) Kit from Thermo Fisher Diagnostics (Hennigsdorf, Germany). The LDH contents of the medium and lysed Phenion FT models, liver spheroids and thyroid follicles were measured using the LDH (IFCC) Kit from Thermo Fisher Diagnostics.

The structures of Phenion FT models and thyroid follicles from all replicate samples in each experiment (except the final Chip3 experiment) were captured using hematoxylin and eosin staining. Photographic images of the liver spheroids and beads containing thyroid follicles placed in replicate circuits for all treatments were also captured.

In the first Chip3 experiment testing topical and systemic application of genistein and daidzein, the structures of all Phenion FT models used were captured using hematoxylin and eosin staining. For all Chip3 experiments, the integrity of the Phenion FT models was confirmed before application and, in the pilot experiment on Days 1, 2, and 5, by measuring the transepithelial electrical resistance (TEER). Since the TEER probe touched the skin model, there was a concern that this procedure could disrupt the skin barrier; therefore, TEER measurements were not conducted during the experiment for the subsequent Chip3 experiments.

### 2.8 Test chemical analysis

Samples were prepared by adding 30 µL acetonitrile plus internal standards (griseofulvin, diazepam and diclofenac) to 60 µL of the standards or samples and mixed vigorously for 10 s. After centrifugation (2,200 × g, 5 min at room temperature), the supernatant was transferred into vials to LC/MS for quantification. Skin samples were extracted by cutting the Phenion FT models into small pieces, stirring on a vortex for 15 min in 1.5 mL of water:acetonitrile (50:50) and then centrifuged. The extraction was performed twice and both supernatants were pooled before analysis.

The HPLC system consisted of a Dionex UltiMate 3000 RS pump and Dionex UltiMate 3000 RS column compartment and Accela Open Autosampler (Thermo Fisher Scientific, United States). Mass spectrometry was performed on a Q-Exactive Plus mass spectrometer (Orbitrap™ technology with accurate mass) equipped with an H-ESI-II (heated electrospray interface) (Thermo Fisher Scientific, United States) connected to a PC running the standard software Xcalibur 4.0.27.19. LC was performed in the gradient mode using 0.1% formic acid in acetonitrile (solvent A) and 0.1% formic acid in water (solvent B); the pump flow rate was set to 600 μL/min. The gradient was as follows: Initial = 5% A; 0.10 min = 5% A; 1.30 min = 97% A; 2.70 min = 97% A; 2.80 min = 5% A; 3.50 min = 5% A. Separation was performed on a Poroshell 120 EC-C18 (2.7u, 100 × 3 mm) analytical column (Thermo Fisher, Schwerte, Germany) with a pre-column C6-Phenyl, 2.6u, 4 × 2.0 mm (Thermo Fisher, Schwerte, Germany) for quantification using gradient elution. The sample injection volume was 12 µL.

Samples were analyzed for genistein, daidzein and MMI using the LC/MS Q-Exactive Plus (Thermo Scientific) instrument in both positive and negative ionization mode with high resolution (70,000) and accurate mass detection (Orbitrap™). The MS was operated in the full scan MS-SIM (m/z: 50–750) mode. The m/z values for the [M-H]- ion of genistein and genistein glucuronide were 269.0453 and 445.0774, respectively. The m/z values for the [M-H]- ion of daidzein, daidzein glucuronide and daidzein sulfate were 253.0501, 431.0982 and 333.0071, respectively. The m/z values for the [M + H]+ ion of MMI was 115.0327. Diazepam was used as ISTD in the positive full scan mode. The specified limit of quantification (LOQ, defined as the concentrations where signal intensity reached 5 times the noise) for genistein, daidzein and MMI were 10, 5 and 15 nM, respectively.

### 2.9 T_4_ and T_3_ quantification

To prepare samples for T_4_ and T_3_ quantification, 10 µL of the internal standard (13C6-L-Triiodo-thyronine (T_3_) and L-thyroxine-13C6 (T_4_)) and 6 µL DMSO/methanol (1:1) were added to 50 µL non-diluted samples of medium. After vortexing, two volumes of acetonitrile containing 0.2% heptafluorobutyric acid were added (100 µL). After mixing and centrifugation (8,000 × rpm, 10 min), 80 µL of the supernatants were subjected to LC-HRMS.

The HPLC system consisted of a Dionex UltiMate 3000 RS pump and Dionex UltiMate 3000 RS column compartment and Accela Open Autosampler (Thermo Fisher Scientific, United States). Mass spectrometry was performed on a Q-Exactive Plus mass spectrometer (Orbitrap™ technology with accurate mass) equipped with an H-ESI-II (positive ionization) (Thermo Fisher Scientific, United States) connected to a PC running the standard software Xcalibur 4.0.27.19. Separation was performed on a Kinetex Phenyl-Hexyl analytical column (2.6 µ, 50 × 2.1 mm from Thermo Fisher, Schwerte, Germany) for quantification using gradient elution using 0.1% formic acid in acetonitrile (solvent A) and 0.1% formic acid in water (solvent B); the pump flow rate was set to 600 μL/min. The gradient was as follows: Initial = 5% A; 0.10 min = 5% A; 0.60 min = 97% A; 1.7 min = 97% A; 1.80 min = 5% A; 3.00 min = 5% A. The injection volume was 3 µL for all samples.

The LOQs for T_4_ and T_3_ quantification in Chip3 medium (with 0.5% BSA) were both 0.3 nM. The limits of detection (LODs, defined as the concentration where the signal intensity was 3 times the noise) for T_4_ and T_3_ quantification in Chip3 medium (with 0.5% BSA) were both 0.05 nM (the only exception was the LOD for T_4_ in the static incubation with genistein and daidzein, which was 0.18 nM). The LOQs for T_4_ and T_3_ quantification in h7H medium were 0.6 and 0.3 nM, respectively. The LOQ for thyroxine 4′-O-beta-D-glucuronide (T_4_-glucuronide) and thyroxine-4′-O-sulfate (T_3_ sulfate) quantification in Chip3 medium was 0.2 and 0.4 nM, respectively. The LOQ for T_4_, T_3_, T_4_-glucuronide and T_3_ sulfate were defined as the concentrations where signal intensity reached 5 times the noise.

T_4_ and T_3_ levels were expressed as 1) nM, 2) pmoles/million cells, 3) a percentage of the initial levels or 4) a percentage of solvent control values at each timepoint. For 3), replicate values for control and treated samples at each timepoint were compared to their mean initial value (on Day 1) and expressed as a percentage. For 4), values were calculated by comparing each replicate from the treated samples with the mean solvent control value for the corresponding timepoint. T_4_:T_3_ ratios were calculated by dividing the T_4_ concentration by the T_3_ concentration present within each separate medium sample. Replicates from all calculations were averaged and the associated error calculated.

### 2.10 Statistical analysis and calculations

Statistical analyses and half-life calculations were conducted using one-way ANOVA or two-way-ANOVA, followed by Dunnett’s multiple comparisons test, using GraphPad Prism version 8.4.3 for Windows, GraphPad Software, San Diego, California United States, www.graphpad.com”. Statistically significant differences with a *p*-value <0.05 are denoted by an asterisk.

## 3 Results

### 3.1 Medium tests in static culture using freshly isolated thyroid follicles

The suitability of tissue-specific media and a mix of these was evaluated using freshly isolated intact thyroid follicles, Phenion FT models and liver organoids. Mixing the liver-thyroid co-culture medium with either skin ALI medium or HepaRG medium did not decrease the viability of the thyroid follicles, Phenion FT models or liver spheroids (see Supplementary Figures 3A–D). T_4_ production was marginally higher when the co-culture medium was mixed with ALI medium and T_3_ production was similar in all three media (Table 1). Halving the number of thyroid follicles from 1,000 to 500 thyroid follicles halved the concentration of both hormones. Notably, the ratio of T_4_:T_3_ produced by the freshly isolated thyroid follicles (2.6–4.8) were similar to the ratio of unbound T_4_:T_3_ reported in the serum of healthy subjects (Table 1).

**TABLE 1:** Comparison of TSH-stimulated T_4_ and T_3_ concentrations and T_4_:T_3_ ratios from clinical samples and in freshly isolated thyroid follicles and thyrocyte-derived thyroid follicles cultured in different formats. Values are a mean ± SD. TSH concentrations were all 1 mU/mL, apart from the second Chip3 experiment, in which 0.1 mU/mL was used. *In vivo* values were from Quinlan et al. (2020).

*In vivo* and *in vitro* hormone levels	T_4_	T_3_	T_4_:T_3_ ratio
	(nM)		
Serum free concentrations *in vivo*	14	4.9	2.9
Static culture of 1,000 freshly isolated thyroid follicles in 0.5 mL 100% Co-culture medium (n = 14)	14.0 ± 3.7	5.4 ± 1.6	2.6 ± 0.2
Static culture of 1,000 freshly isolated thyroid follicles in 0.5 mL Co-culture:ALI 50:50 mix (n = 14)	21.2 ± 5.7	5.5 ± 1.8	3.9 ± 0.3
Static culture of 1,000 freshly isolated thyroid follicles in 0.5 mL Co-culture:ALI 70:30 mix (n = 14)	22.3 ± 8.1	7.4 ± 3.3	3.2 ± 0.6
Static culture of 500 freshly isolated thyroid follicles in 0.5 mL 100% Co-culture medium (n = 14)	7.1 ± 2.2	2.5 ± 0.9	2.9 ± 0.4
Static culture of 500 freshly isolated thyroid follicles in 0.5 mL Co-culture:ALI 50:50 mix (n = 14)	8.8 ± 3.1	1.8 ± 0.7	4.9 ± 0.3
Static culture of 500 freshly isolated thyroid follicles in 0.5 mL Co-culture:ALI 70:30 mix (n = 14)	11.9 ± 5.4	3.0 ± 1.4	4.0 ± 0.4
Static culture thyrocyte-derived thyroid follicles using Method 3 in 0.5 mL medium, Donor 1 + 4, n = 9 samples from solvent controls over Days 1–4	1.1 ± 0.2	0.44 ± 0.10	2.4 ± 0.7
Chip3 pilot using thyrocyte-derived thyroid follicles from Donor 1 using Method 3 in 1 mL medium (mean of solvent controls on Days 1–2, n = 3 chips)	0.93 ± 0.23	0.23 ± 0.06	4.1 ± 1.0
Chip3 experiment 1 thyrocyte-derived thyroid follicles from Donor 2 using Method 3 in 1 mL medium (mean of solvent control on Days 1–4, n = 3 circuits)	1.4 ± 0.5	0.35 ± 0.13	3.9 ± 0.5
Chip3 experiment 2 thyrocyte-derived thyroid follicles from Donors 4 and 6 using Method 3 in 1 mL medium (mean of solvent controls on Days 1–2, n = 7 circuits)	0.5 ± 0.19	0.36 ± 0.10	1.54 ± 0.36

### 3.2 Thyrocyte-derived thyroid follicle development

The supply of fresh human thyroid tissue stopped after the initial experiment; therefore, subsequent experiments were performed using cryopreserved human thyrocyte-derived thyroid follicles. As a result, the mix of medium was changed from liver-thyroid co-culture medium + skin ALI medium to h7H medium + skin ALI medium.

#### 3.2.1 Pilot study in Chip3 using thyrocyte-derived thyroid follicles from method 1

**FIGURE 2:**
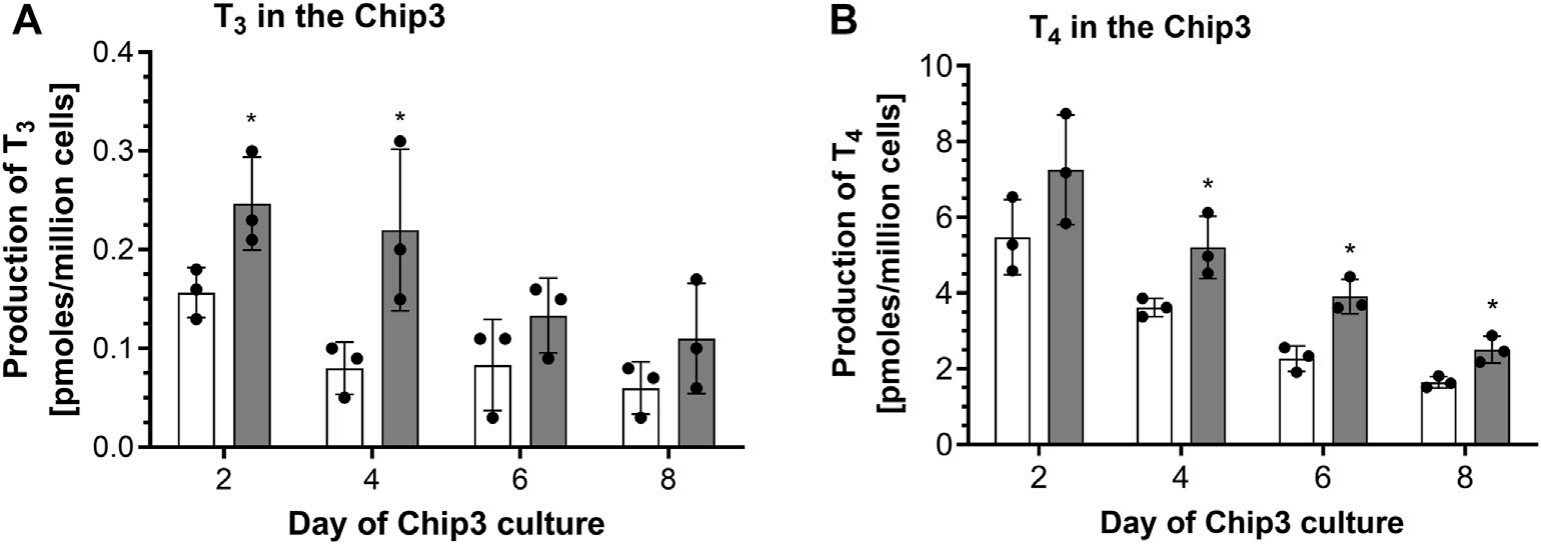
Basal and TSH-stimulated T_3_ **(A)** and T_4_ **(B)** hormone production in the Chip3 incubations from Donor 1 over time (Method 1 thyroid follicles). Basal (white bars) and 1 mU/mL TSH-stimulated (grey bars) hormone production is expressed as a mean ± SEM of pmoles/million cells, n = 3. The black circle symbols are the individual data points. Statistically significant differences (*p* < 0.05) between basal and TSH-stimulated hormone levels are denoted with an asterisk.

The first time thyrocyte-derived thyroid follicles were tested in the Chip3, it was assumed that more cells in the beads would lead to higher hormone production. Therefore, the method of (Kühnlenz et al., 2022) was modified to entrap 150,000 thyrocytes from Donor 1 (i.e., Method 1) instead of freshly isolated thyroid follicles. When this method was used, basal and TSH-stimulated hormone production in the Chip3 decreased over time (Figure 2). This was in contrast to the basal and TSH-stimulated T_3_ levels in static cultures of thyroid follicles from the same donor, which increased consistently over time (Supplementary Figure S4). In addition, T_3_ production in the Chip3 was much lower than that from thyrocyte-derived thyroid follicles from the same donor but cultured according to Method 2 under static conditions (Supplementary Figure S4). It was not possible to measure T_4_ in static cultures prior to adding the thyroid follicles to the Chip3 due to high background levels in the FCS used (from Sigma), despite it being charcoal-stripped. All further experiments with FCS were from a different supplier (Gibco) to avoid this issue. Despite the lower hormone levels in the Chip3, there was a statistically significant stimulation of T_3_ by 1 mU/mL TSH (Figure 2A). Similarly, T_4_ production by thyroid follicles in the Chip3 was also stimulated by TSH (which were measurable since BSA was used in the Chip3 instead of FCS) (Figure 2B). A mean T_4_:T_3_ ratio of 4.2-fold was observed in the Chip3 in the presence of 1 mU/mL TSH (Table 1).

Possible reasons for the observed decrease in hormone levels over time in the Chip3 were investigated. These included 1) metabolism by the liver spheroids, 2) binding to proteins in the medium, 3) loss of viability of the organoids and 4) sub-optimal culture of the thyroid follicles. The first three reasons were ruled out by 1) testing the medium for T_4_ and T_3_ metabolites—none were detected (i.e., below the LOD), 2) incubating T_4_ and T_3_ with BSA or thyroglobulin for 2 h—there was no loss in the mass balance, and 3) analyzing the media for glucose, lactate, LDH and albumin—none indicated a loss of viability over time (data not shown). The fourth reason was investigated in a separate experiment in which different culture methods were compared—as described in the next section.

In static cultures, after the application of a single dose of MMI on Day 10, T_3_ production by non-stimulated and TSH-stimulated thyroid follicles was decreased by 21%–22% on Day 12, and by 48% on Day 14. By contrast, in the Chip3 model, a single application of MMI on Day 12 did not statistically significantly affect T_3_ production by non-stimulated or TSH-stimulated thyroid follicles (data not shown).

#### 3.2.2 Optimization of thyroid follicle culture for Chip3: Comparison of T_4_ and T_3_ production and morphology

Two notable differences between the freshly isolated thyroid follicles and the thyrocyte-derived thyroid follicles using Method 1 were observed. Firstly, freshly isolated thyroid follicles were much larger than follicles formed from isolated thyrocytes (Supplementary Figure S5). Despite this difference, the thyrocyte-derived thyroid follicles formed aggregates of cells with the clear presence of lumen, typical for thyroid follicles. Secondly, static cultures of thyrocyte-derived thyroid follicles produced ∼10-fold lower concentrations of hormones compared to freshly isolated thyroid follicles (Table 1). Although the levels of hormones were different in freshly isolated thyroid follicles and thyrocyte-derived thyroid follicles both produced similar ratios of T_4_:T_3_.

**FIGURE 3:**
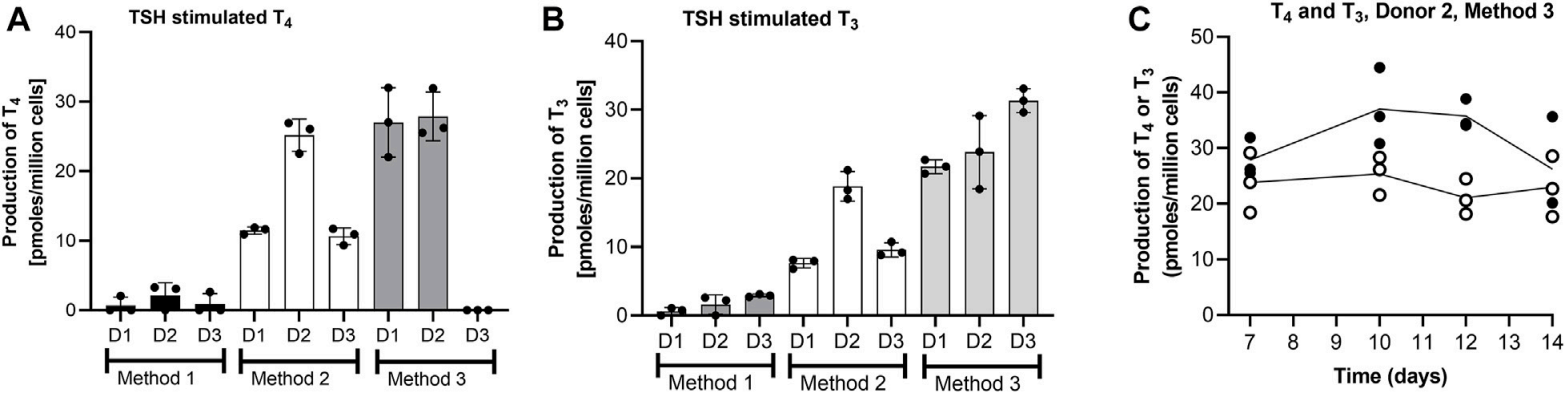
T_4_ and T_3_ production by thyrocyte-derived thyroid follicles produced using different culture methods **(A,B)**, and T_4_ (black symbols) and T_3_ (white symbols) production over time by thyroid follicles produced according to Method 3 using thyrocytes from Donor 2 **(C)**. Method 1 was a Matrigel-entrapment method with 150,000 cells per well, Method 2 was according to Deisenroth et al. (2020), and Method 3 was an adaptation of Method 2 with an overlay of Matrigel. Values in A and B are expressed as a mean ± SEM of pmoles/million cells, n = 3, with the black circle symbols denoting individual data points. Values in C are individual values from three circuits are shown with the connecting line denoting the mean.

The effect of the thyrocyte culture method on the T_4_ and T_3_ production by thyroid follicles on Day 7 of culture is shown in Figures 3A,B. Since the number of cells were different, the values here (and elsewhere) were normalized to the number of cells seeded. Method 3 resulted in the highest production of both hormones from one or more donors. The lowest T_4_ and T_3_ production was observed when single cells were entrapped in Matrigel (Method 1), even though the cell number was 10-fold higher than for Methods 2 and 3. The highest donor-variation was observed for T_4_ production, with Donor 2 producing the highest levels when cultured in all three methods, whereas Donor 3 produced negligible amounts of T_4_ when cultured using Method 3. Based on these results, Donor 3 was ruled out as a source of cells for the Chip3 experiment, since Method 3 was optimal for the practical application in the chip circuit. Figure 3C shows the TSH-stimulated T_3_ and T_4_ production in static incubations with thyroid follicles from Donor 2 using Method 3 between Days 7 and 14. T_3_ levels were relatively stable over time; however, T_4_ production decreased in the last 2 days from 35.8 ± 1.5 to 26.2 ± 4.8 pmoles/million cells. Donors 1 and 3 were only incubated up to Day 11 but levels at this timepoint were similar to those on Day 7 (data not shown). Based on these results, the duration of the culture of thyroid follicles for the first main Chip3 experiment was limited to 10 days.

### 3.3 Concentration-response curves in static incubations

**FIGURE 4:**
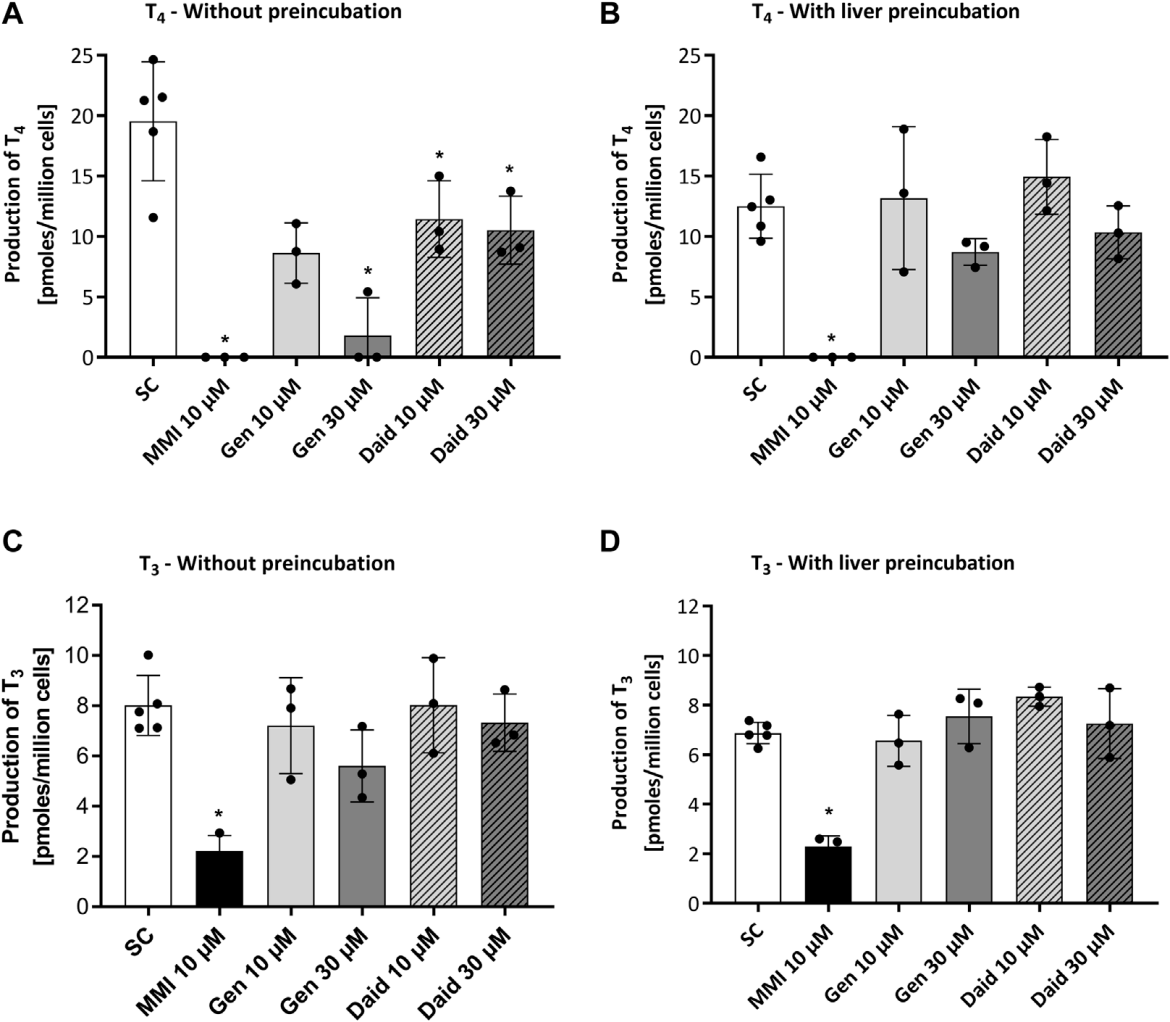
Effect of MMI, genistein (Gen) and daidzein (Daid) on TSH-stimulated T_4_ **(A,B)** and T_3_ **(C,D)** production by thyrocyte-derived thyroid follicles after daily application without **(A,C)** and with **(B,D)** pre-incubation for 24 h with liver organoids. TSH-stimulated T_4_ and T_3_ values, expressed as a mean ± SEM of pmoles/million cells, n = 3, are shown after four and three applications, respectively. The black circle symbols are the individual data points. Statistically significant differences (*p* < 0.05) between test chemical-treated and solvent control-treated hormone levels are denoted with an asterisk.

The aim of the main Chip3 experiments was to compare effects of topical doses genistein and daidzein with systemic doses which were known to inhibit T_4_ and T_3_ levels. Before this was run, it was important to confirm that both chemicals inhibited hormone production by thyroid follicles cultured according to the selected culture method (Method 3) and medium (50:50 h7H:ALI medium). In addition, the impact of the metabolism of the chemicals by liver organoids prior to incubation with the thyroid follicles was assessed. Therefore, a study was conducted in static cultures, in which the effect of a range of repeated daily doses of genistein, daidzein and MMI on TSH-stimulated T_4_ and T_3_ production was evaluated over 4 days (thus mimicking to some extent the dosing application scenario in the Chip3 experiments). TSH-stimulated T_4_ production was decreased by repeated applications and increasing concentrations of genistein and daidzein (Supplementary Figures S6A, B). Repeated application of 10 µM MMI completely inhibited T_4_ production by Day 4% and 72% of T_3_ production by Day 3 (T_3_ was not present in any samples on Day 4) (Figure 4 and Supplementary Figures S7A, B). T_4_ and T_3_ inhibition by genistein was higher than daidzein at the same concentrations, indicating a higher inhibitory potency of genistein (Figures 4A–C). The inhibitory effect of all chemicals was more profound on T_4_ than on T_3_ production, which resulted in a lower T_4_:T_3_ ratio at concentrations of genistein and daidzein higher than 1 µM (Supplementary Figures S6C, D). Based on the concentration-response curves, T_4_ inhibition IC_50_ values for genistein and daidzein were 54 µM and 152 μM, respectively. It was not possible to determine the IC_50_ values for T_3_ since the highest test concentration of genistein (30 µM) did not exceed 30% inhibition.

After a 24 h pre-incubation with liver organoids before adding to thyroid follicles, 50%–60% of daidzein and up to 70% of genistein were metabolized (see Supplementary Material S2), which decreased the extent of T_4_ and T_3_ inhibition (Figures 4B–D). By contrast, MMI was not metabolized by the liver organoids (data not shown) and the extent of inhibition T_4_ and T_3_ was unaltered. Notably, the production of T_4_ and T_3_ in the presence of the liver incubates with the solvent control was lower than when test chemicals were added in fresh medium. This could indicate that the thyroid follicles are sensitive to changes in the “spent” medium composition due to the presence of the liver spheroids. Despite the lower T_4_ and T_3_ levels in the presence of preincubated media, the % inhibition by the test chemicals could still be compared to the levels in the solvent control samples, which were incubated under the same conditions.

Information from this study was used to set the systemic doses of genistein and daidzein in the Chip3 and ensure that sufficient test chemical was present to inhibit hormone production (see methods Section 2.6.3 *Chip3 - dose selection for genistein and daidzein*).

### 3.4 Chip3 incubations testing genistein and daidzein

#### 3.4.1 First Chip3 experiment

**FIGURE 5:**
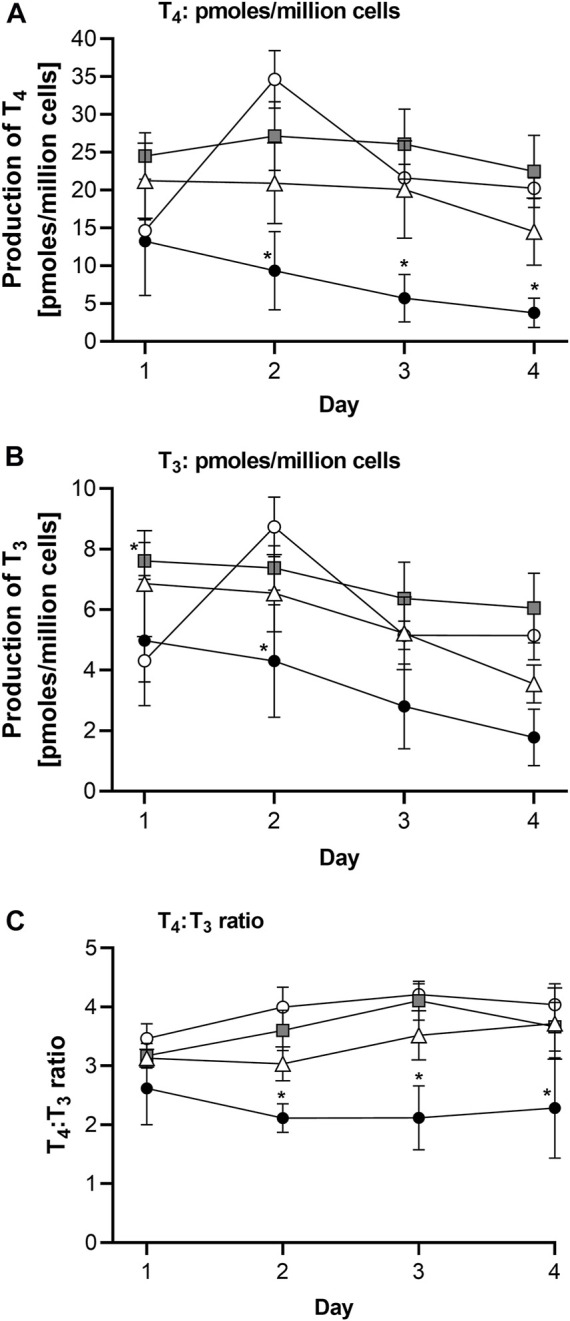
TSH-stimulated T_4_ **(A)**, T_3_ **(B)** levels and T_4_:T_3_ ratios **(C)** in the Chip3 over 4 days after repeated daily topical applications of a lotion formulation containing 1.55 μg/cm^2^ genistein (white triangles) or 2.35 μg/cm^2^ daidzein (grey square symbols) or the lotion formulation alone (white circles). The positive control was 10 µM MMI (black circles), applied systemically daily at each medium change. T_4_ and T_3_ levels in **(A)** and **(B)** are expressed as a mean ± SEM of pmoles/million cells, n = 5 circuits. Values that are statistically significantly (*p* < 0.05, 2-way ANOVA) lower than solvent control-treated hormone levels are denoted with an asterisk.

The effects of repeated topical applications of 1.55 μg/cm^2^ genistein and 2.35 μg/cm^2^ daidzein in a lotion formulation on T_3_ and T_4_ in the Chip3 over 4 days were investigated (Figure 5). These topical doses were expected not to have any effects on T_4_ or T_3_ compared to systemic applications of LOECs. None of the treatments tested caused any changes in viability parameters compared to solvent control conditions (see Supplementary Figure S8). T_4_ and T_3_ concentrations in solvent control treated Chip3 circuits peaked on Day 2 and still exceeded initial values at the end of the experiment (Day 4; 139% and 120% of the initial concentrations, respectively). Repeated topical applications of genistein and daidzein did not statistically significantly alter T_4_ and T_3_ concentrations compared to solvent control values; however, unlike solvent control-treated circuits, they did decrease over time to 64% (T_4_) and 57% (T_3_) of their initial values after genistein treatment (which was statistically significant) and to 88% (T_4_) and 79% (T_3_) of the initial concentration after daidzein treatment (Supplementary Figure S9). In addition, the T_4_:T_3_ ratio was generally lower after genistein treatment compared to solvent control ratios (Figure 5C). Both these observations suggested that the doses selected did cause a change in the hormone production and could be considered to be the lowest observed effect level (LOEL) doses.

Unfortunately, in this first experiment, the solvent used for the systemic application of genistein and daidzein (ethanol at a final concentration of 2% v/v) was toxic to the thyroid organoids, evident as a complete loss of T_4_ and T_3_ production, making it impossible to interpret this application scenario. This loss of viability of the thyroid follicles was not evident in the glucose, lactate or LDH measurements, which were all comparable to solvent control values (Supplementary Figure S8).

#### 3.4.2 Second Chip3 experiment

Since the first Chip3 experiment could not confirm a lack of an effect of genistein and daidzein after topical application and the unexpected toxicity to thyroid follicles by systemically applied ethanol, a second experiment was conducted. The main changes compared to the first experiment were: 1) the solvent for systemic application of genistein and daidzein (100% DMSO was used at a final concentration of 0.1% v/v instead of ethanol), 2) the TSH concentration was reduced to 0.1 mU/mL based on additional static incubations indicating 1 mU/mL was too high (data not shown), 3) a longer test chemical incubation period of 5 days (to capture potentially latent effects) 4) collection of all media and organoids on Day 5 (i.e., the end of the incubation in the Chip3), for the measurement of the distribution of test chemicals and their metabolites, and 5) excluding the topical application of genistein and testing two doses of daidzein, one at the LOEL and a second at an estimated safe dose. While it would have been preferable to test multiple doses of both chemicals, it was not practically possible—indeed the maximum number of chips were used in both experiments (35 chips). Therefore, we focused on the effects of topical doses of daidzein compared with the systemic application of the positive control chemical, MMI, and LOECs of genistein and daidzein. None of the treatments tested caused any changes in viability parameters compared to solvent control conditions (see Supplementary Figure S10). The morphology of the skin, liver and thyroid follicle organoids after selected dosing scenarios are shown in Supplementary Figure S11.

**FIGURE 6:**
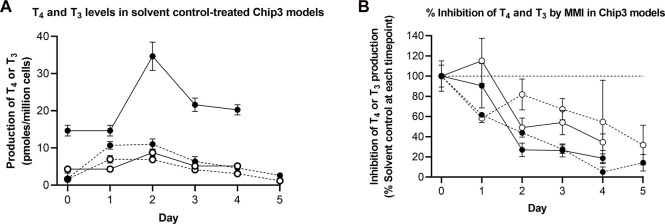
TSH-stimulated T_4_ and T_3_ levels in solvent control-treated Chip3 models **(A)** and inhibition of TSH-stimulated T_4_ and T_3_ production by MMI measured in the first (solid lines) and second (dotted lines) main Chip3 experiments **(B)**. TSH-stimulated T_4_ (closed symbols) and T_3_ (open symbols) concentrations are expressed as a mean ± SEM of pmoles/million cells, n = 3–4 circuits. For the inhibition by MMI, values were calculated by comparing each replicate from the treated samples with the mean solvent control value for the corresponding timepoint. The horizonal dotted line in **(B)** represents 100% of solvent control).

In this experiment, notably lower concentrations of T_4_ were observed in solvent control samples compared to those in the first experiment, whereas the concentrations of T_3_ were similar in both experiments (Table 1; Figure 6A). The concentration of both hormones decreased in solvent controls over time, such that by Day 5, T_4_ and T_3_ were 37% and 18% of the initial concentrations, respectively. To validate the results from the second experiment, the effect of the positive control, MMI, was expressed as a percentage of the solvent control at each timepoint and then compared between the two experiments (Figure 6B). In both experiments, MMI resulted in a time-dependent decrease of T_4_, and T_3_ - down to 19% and 37% of the solvent control, respectively, in the first experiment (by Day 4) and by 5%–14% and 32%–55% of the solvent control, respectively, in the second experiment (by Days 4–5). Importantly, the profile of hormone inhibition was similar; therefore, for this second experiment, all data were expressed as a % of the solvent control at each timepoint.

**FIGURE 7:**
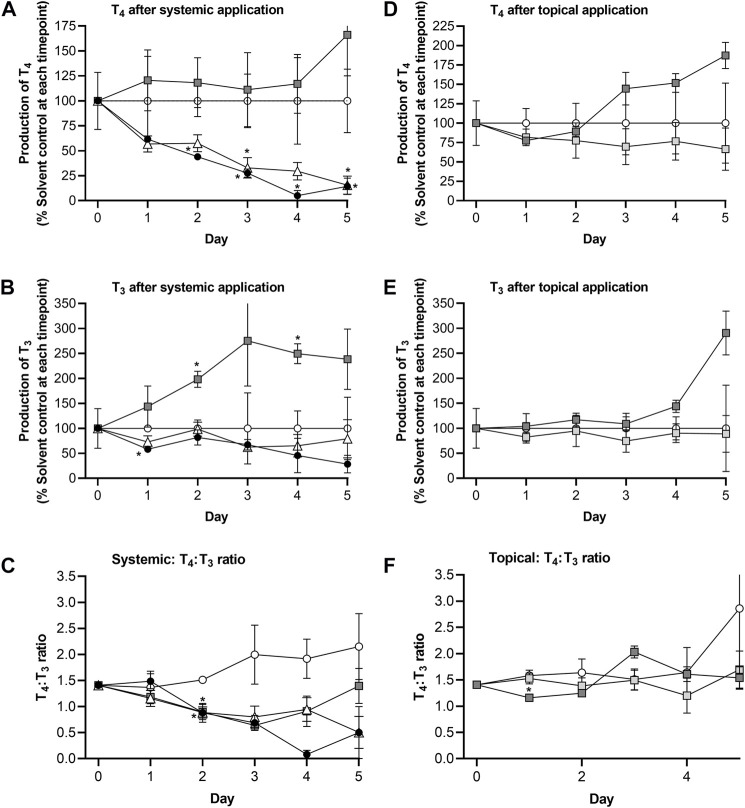
Comparison of route effects using the skin-liver-thyroid Chip3 model: Effect of repeated systemic application of solvent control (open circles), 25 µM genistein (white triangles), 58 µM daidzein (dark grey squares) and 10 µM MMI (black circles) on TSH-stimulated T_4_ levels **(A)**, T_3_ levels **(B)** and T_4_:T_3_ ratios **(C)**, and effect of repeated topical application of solvent control (open circles, a predicted LOEL dose (dark grey squares) and a “safe dose” (light grey squares) of daidzein on TSH-stimulated T_4_ levels **(D)**, T_3_ levels **(E)** and T_4_:T_3_ ratios **(F)**. The values on Day 0 in A, B, D and E are a mean ± SEM of samples from all Chip3 circuits prior to treatment. Values are a mean ± SEM of 3-5 circuits. Values that are statistically significantly (*p* < 0.05, 2-way ANOVA) different compared to solvent control-treated hormone levels are denoted with an asterisk.

Repeated systemic application of 25 µM genistein resulted in a time-dependent decrease of T_4_ down to 16% of the solvent control values by Day 5, the profile of which was similar to that after MMI treatment (Figure 7A). Like MMI, genistein also decreased T_3_ concentrations but to a lesser extent than T_4_ (wn to 63%–80% of solvent control treated circuits (Figure 7B)). This lowered the T_4_:T_3_ ratio from 1.4 on Day 0 to 0.47 on Day 5. Repeated systemic application of 58 µM daidzein increased both the T_4_ and T_3_ concentrations by different extents (Figures 7A, B), resulting in a lowered the T_4_:T_3_ ratio comparable to that caused by genistein and MMI over the first 3 days. However, the T_4_:T_3_ ratio appeared to recover after treatment with daidzein over the remaining 2 days. This recovery was not observed after exposure to MMI or genistein (Figure 7C).

The topical LOEL dose caused both hormones to increase compared to solvent control concentrations - up to 187% (T_4_) and 291% (T_3_) of solvent control values by Day 5. Despite these increases, the T_4_:T_3_ ratio in LOEL dose-treated incubations was unaltered over time. Importantly, repeated topical application of the estimated “safe dose” (0.235 μg/cm^2^) of daidzein affected neither T_3_ nor T_4_ concentrations over 5 days (Figures 7D, E). As a result, the T_4_ and T_3_ ratio was also unaltered (Figure 7F).

**FIGURE 8:**
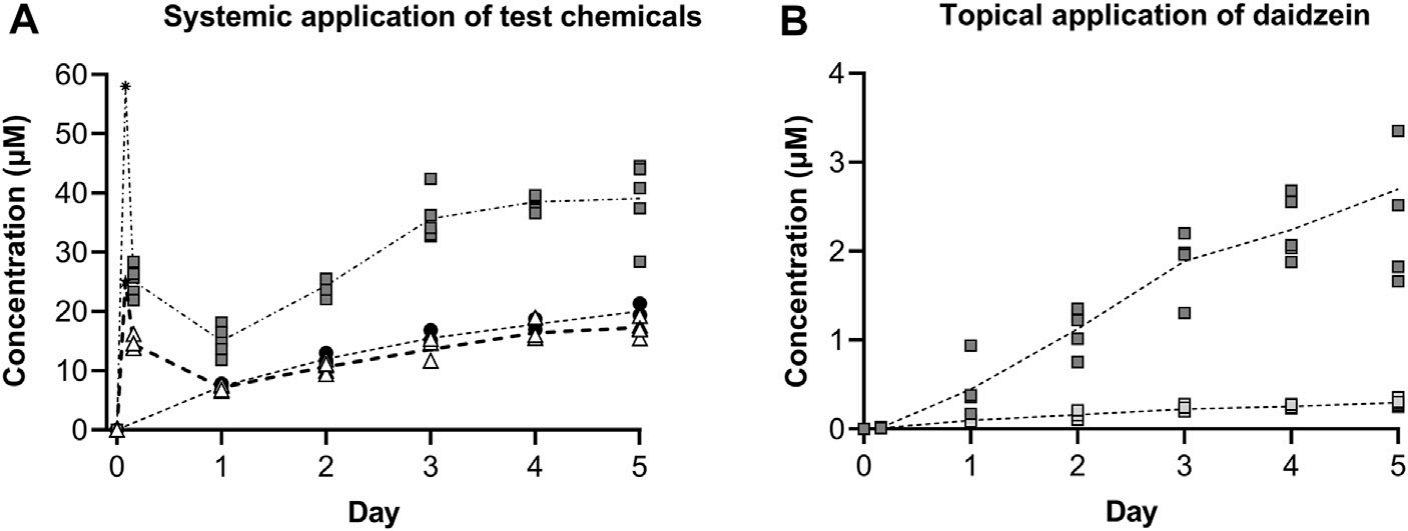
Concentrations of test chemicals in the medium after repeated systemic application of 25 µM genistein (black circles), 58 µM daidzein (grey squares) and 10 µM MMI (white triangles) **(A)**, and repeated topical application of a predicted LOEL dose (dark grey squares) and a “safe dose” (light grey squares) of daidzein **(B)**. Individual values from 3–5 circuits are shown with the connecting line denoting the mean. For the systemic application of 25 µM genistein and 58 µM daidzein, the target initial concentration at time zero has been added (denoted by a star and asterisk, respectively.

The concentrations of the test chemicals in the medium at each timepoint are shown in Figure 8. It should be noted that the medium concentration was halved at each timepoint due to replacing half of the medium to sustain the organoid culture; therefore, the symbols denote “trough” concentrations and are connected using dotted lines. Medium samples for the single early timepoint (at 4 h) were collected for systemic applications of genistein and daidzein, which showed initially high concentrations and that ∼40% of genistein (initial was 25 μM, denoted by a star in Figure 8) and ∼57% of daidzein (initial was 58 μM, denoted by an asterisk in Figure 8) had already been metabolized by this time. The metabolites of genistein and daidzein were also monitored and detected but since their formation was associated with decreased T_4_ and T_3_ inhibition in the static incubations (i.e., they are detoxification pathways), they are not shown. The concentrations of genistein increased over time and were comparable to those of MMI (which was not metabolized, data not shown). The concentrations of daidzein also increased over time but, unlike genistein or MMI, there was noticeable visible precipitation on the liver spheroids after systemic application (Supplementary Figure S11).

Topical application of both the LOEL dose and the “safe dose” resulted in detectable daidzein concentrations in the circuits (0.45 ± 0.13 and 0.01 ± 0.00 µM, respectively, on Day 1, Figure 8B). These concentrations were lower than those expected after systemic administration of the same doses to 1 mL circuit volume.

LOEL dose = 2.35 μg/cm^2^ = 1.41 µg/skin model = 5.55 nmoles/skin model → 5.55 nmol/mL circuit = 5.55 µM
“Safe dose” = 0.235 μg/cm^2^ = 0.141 µg/skin model = 0.555 nmoles/skin model → 0.56 nmol/mL circuit = 0.56 µM

After repeated topical application, the concentrations increased to 2.70 ± 0.47 µM and 0.30 ± 0.02 µM for the LOEL dose and “safe dose”, respectively.

**FIGURE 9:**
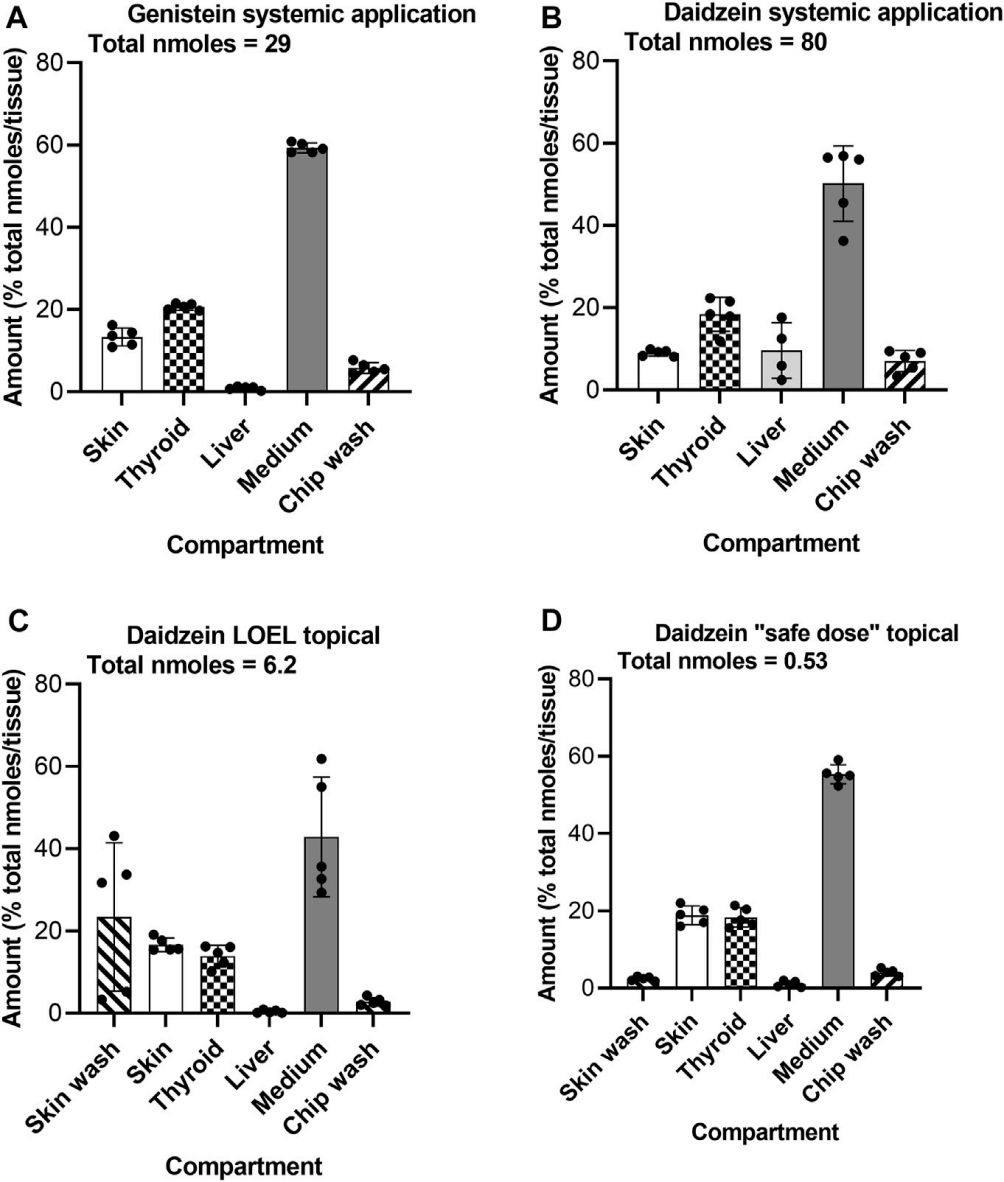
Distribution of genistein **(A)** and daidzein **(B–D)** in the Chip3 circuit and organoids on Day 5 after systemic **(A,B)** and or topical **(C,D)** application. Values are expressed as a % of the total number of nmoles in the compartments (denoted under the title under each graph), mean ± SEM of 5 circuits. The black circle symbols are the individual data points.

At the end of the incubation, the presence of test chemical and metabolites was analyzed in the different organoids and Chip3 compartments to show their distribution and to confirm that the thyroid follicles were exposed to them. On Day 5, the majority (43%–55% of the total nmoles) of test chemical was in the medium (Figure 9). Due to some precipitation of daidzein on liver spheroids after systemic application, ∼10% of this chemical was recovered from the liver compartment (Figure 9B). Importantly, 14%–20% of the test chemicals were recovered from the thyroid follicles, regardless of the route of application or dose applied. Only 2% of the topical “safe dose” of daidzein remained on the skin surface 24 h after its last application, indicating that most had entered the skin and the systemic circulation. Approximately 20% of the LOEL dose (which was 10-fold higher than the “safe dose”) remained on the skin surface, suggesting that absorption of this dose was not complete. Analysis of the metabolites showed a similar distribution, with the exception that no metabolites were recovered in the skin washes or chip washes (data not shown).

## 4 Discussion

### 4.1 Justification of the skin-liver-thyroid model

The aim of this study was to develop a skin-liver-thyroid Chip3 model which could be used in a consumer-relevant safety assessment for topically applied chemicals. The Chip3 model is closer to the *in vivo* situation than traditional 2D monocultures since the test chemicals were topically applied to human skin in a body lotion and systemic effects related to the thyroid were measured. The main molecular initiating events for chemically-induced thyroid activity involve either the thyroid gland directly (direct thyroid toxicity) or target thyroid hormone catabolism through the induction of hepatic enzymes (indirect thyroid toxicity) (Noyes et al., 2019). This model allows the evaluation of both factors at the same time, although the effects of neither genistein nor daidzein on liver spheroid metabolizing enzymes were analyzed in this study. As safety assessments are focused on human health, an added advantage of the model was that all the cell types used were human. This is especially relevant for evaluating thyroid effects since rodents are reported to have an increased sensitivity to impairments in thyroid hormone homeostasis (Dietrich et al., 2015). Moreover, use of human cells obviates the requirement for inter-species safety assessment factors, though other factors for *in vitro:in vivo* extrapolations as well as safety factors to warrant consumer-protective approaches may be required and were used in this study for e.g., the calculation from LOECs to NOECs.

An advantage of the Chip3 model is that the kinetics of the test chemical (and metabolites) in the circuit can be directly linked to the toxicodynamic effects over time. For example, the profiles of the inhibition of T_4_ production by MMI and genistein and the concentrations of both chemicals measured at each timepoint were similar. This suggests that repeated systemic doses of 25 µM genistein was equipotent with repeated systemic doses of 10 µM MMI. The difference between the two chemicals is that genistein was metabolized and MMI was not. Repeated systemic application of daidzein reached a steady state concentration of ∼40 µM in the Chip3 by Day 3 (2-fold higher than genistein or MMI). Interestingly, unlike genistein and MMI, this chemical increased thyroid hormone levels and at the same time, decreased the T_4_:T_3_ ratio. This observation was also in contrast to the effect of daidzein in static cultures of thyroid follicles (Supplementary Figure S6D). While the reason for this needs more investigation, it does highlight the difference in the effects of a chemical on T_4_ and T_3_ between static thyroid follicle incubations and MPS models combining thyroid follicles with skin and liver organoids.

### 4.2 Technical aspects of the Chip3 design

#### 4.2.1 Replacement of freshly isolated thyroid follicles

An immediate hurdle in this study was the lack of a reliable supply of fresh human thyroid follicles. This was overcome by generating thyroid follicles using cryopreserved primary human thyrocytes, which had been isolated in two of our laboratories for exploratory use. The use of cryopreserved cells allowed easy scheduling of the experiments and the pre-qualification of thyrocytes from healthy donors before using them in the Chip3, which is not possible using freshly isolated thyroid follicles. Thyrocyte batches that were qualified for use produced similar concentrations of hormones, indicating a low donor-variability; however, we tried to overcome potential donor variation by using thyrocytes from two donors in the final Chip3 experiment. Primary cells were preferred over the use of immortalized cell lines to avoid their associated disadvantages e.g., becoming dedifferentiated and non-responsive to TSH over extensive passaging (van Staveren et al., 2007). The formation of thyroid follicles in culture requires careful 3D culture techniques to be able to express key proteins such as thyroglobulin and exhibit a polarized form (Kraiem et al., 1991; Mauchamp et al., 1998; Kusunoki et al., 2001; Bernier-Valentin et al., 2006; Toda et al., 2011; Saito et al., 2018; Deisenroth et al., 2020). We based the culture of thyrocytes in our studies on the method of Deisenroth et al. (2020), in which thyrocytes were able to self-organize into follicle-like structures when placed on top of Matrigel. Deisenroth et al. reported mean ranges of 8,741–9,311 pg/mL of T_4_ and 2,700–3,792 pg/mL of T_3_ being produced by thyrocyte-derived thyroid follicles after stimulation with 1 mU/mL TSH, which equate to 75–80 pmoles/million cells T_4_ and 27.6–38.9 pmoles/million cells T_3_. Using the same method (Method 2), the production of thyroid hormones in this study were somewhat lower although in the same order of magnitude, with 10.7–25.2 pmoles/million cells T_4_ and 7.6–18.8 pmoles/million cells T_3_. Although not directly comparable, the concentrations of T_4_ and T_3_ produced by freshly isolated thyroid follicles were ∼10-fold higher than thyrocyte-derived thyroid follicles, which could be attributed to the smaller size and number of fully functional follicles present in the latter. Despite the lower thyroid hormone production by the thyrocyte-derived thyroid follicles, they were still considered to be high enough for the purposes of the case study, especially since the ratio of T_4_ to T_3_ known to occur *in vivo* (Quinlan et al., 2020) was also exhibited by these thyroid follicles. While the method of Deisenroth et al. (2020) was shown to be suitable for medium-throughput assays, it was not suitable for use in the Chip3 since it was designed for multi-well plates, whereas the Chip3 compartments need spheroids or beads of cells/tissues which cannot detach and move around the circuit. Therefore, a method in which thyroid follicles could be retained in beads was developed. Interestingly, increasing the cell number and entrapping thyrocytes in Matrigel did not increase the production of T_4_ and T_3_, moreover, there was lower production per million cells than cultures with 10-fold lower cell numbers. TSH-stimulated thyroid hormone production by thyroid follicles from two of the three donors tested was increased to ∼30 pmoles/million cells by adding a Matrigel overlay to the self-formed thyroid follicles seeded on top of Matrigel, which was maintained in static culture over 10–12 days. Since this method (Method 3) was also more practically applicable to the Chip3 model (the beads of thyroid follicle-containing Matrigel were easily scooped out of the round-bottomed wells and placed into the Chip3 compartment), this was adopted for the main Chip3 experiments.

#### 4.2.2 Chip3 medium optimization

Having identified a method for thyroid follicles formation, the Chip3 medium was successfully modified to exchange FCS with BSA, which was more suited to the Phenion FT models and liver spheroids. Another advantage of BSA was that this resulted in lower background levels of T_4_ and T_3_ in the medium and lower LOQs for the analytical method. While the Chip3 medium was modified to optimize thyroid follicles function, this should not be at the expense of the viability of other organoids in the Chip3. The h7H medium contains phenol red, which is less suited to HepaRG cells, therefore alternative phenol red-free thyroid medium and different ratios of the thyroid and ALI medium could be tested in future studies using these cells.

Another change to the medium which was instigated in the final Chip3 experiment was the lowering of the TSH concentration from 1 to 0.1 mU/mL. The reason for this was because concentration response curves in separate static thyrocyte-derived thyroid follicle experiments exhibited a bell-shaped curve with concentrations greater than 0.1 mU/mL resulting in lower T_4_ values (data not shown). However, although the lower TSH concentration had minimal effects on T_3_ levels in the Chip3, T_4_ production was 1.5- to 2-fold lower, and levels gradually decreased after Day 2. In addition to the differing results from static and dynamic incubations regarding the optimal TSH, there was also a difference in the inhibition of hormone synthesis by MMI. T_4_ and T_3_ production in static incubations were inhibited by a single application of MMI, whereas sustained inhibition by MMI in the Chip3 required repeated dosing (possibly due to recovery of hormone production in the latter). Therefore, future optimization steps for the Chip3 will be conducted in the chips, rather than basing the experimental design on findings from static incubations.

#### 4.2.3 Liver spheroid models

Future studies could explore the use of primary human hepatocytes to form liver spheroids. These better represent the metabolism of thyroid hormones than HepaRG cells (which did not metabolize T_4_ or T_3_ in this study) and are not sensitive to the presence of phenol red nor high concentrations of ethanol (Easterbrook et al., 2001). Pooled batches of human hepatocytes could also be used to address donor variation or, alternatively, incubations can be carried out using thyrocytes and hepatocytes from the same donor.

#### 4.2.4 Solvent controls

In the first Chip3 experiment comparing topical and systemic applications, ethanol was used as the solvent for the systemic application of genistein and daidzein at a final concentration of 2% v/v. This was toxic to the thyroid organoids, evident as a complete loss of T_4_ and T_3_ production, but not to the skin or liver organoids (since there was no alteration in glucose, lactate or LDH measurements compared to solvent control values). In retrospect, it may have been expected that this concentration of ethanol would cause a loss of hormone production, since it is reported to directly suppress thyroid function *in vivo* due to cellular toxicity (Balhara and Deb, 2013). Therefore, future studies will evaluate which solvents can be used for *in vitro* assays using thyrocyte-derived thyroid follicles and, importantly, the highest concentrations which can be used. The endpoint best suited to detecting a loss of thyroid follicle viability in the Chip3 is the production of T_4_ and T_3_, while other markers measured in the Chip3 are more reflective of skin and liver spheroid viability.

#### 4.2.5 PBPK model for dose setting

The topical doses of genistein and daidzein for the main experiments were set using a whole body PBPK model to predict a dose that would result in a specific target plasma concentration. When applied to the Chip3 model, the resulting circuit concentrations after repeated topical applications of daidzein were much higher (2.70 ± 0.47 µM) compared to the intended target plasma concentration of 93 nM. This indicated that the compartments considered in the whole-body PBPK model are not representative of the Chip3 circuit and were not optimized to the scaled-down size of the Chip3 circuit (and distribution in the body). To implement a PBPK model for selecting a safe dose in the Chip3 experiments, further refinements are needed to reflect the Chip3 conditions and the associated parameters used for building the models. For the current study, more empirical estimations based on the amount applied, dermal penetration and the circuit volume (1 mL in this case) may have been more suitable for dose setting in the absence of a Chip3-specific PBK model.

#### 4.2.6 Test chemical inhibition of thyrocyte-derived thyroid follicle hormone production

Before testing chemicals in the Chip3, confirmation was needed that they could indeed inhibit T_4_ and T_3_. Therefore, static incubations of thyrocyte-derived thyroid follicles treated daily with genistein, daidzein and MMI confirmed that T_4_ and T_3_ were both inhibited by all three chemicals and that the biological activity of daidzein was lower than genistein. Moreover, the IC_50_ values for genistein and daidzein inhibition of T_4_ (54 µM and 152 μM, respectively) were comparable to those derived from the thyroid peroxidase inhibition assays (54 µM and 73 μM, respectively), thus confirming the suitability of the thyroid follicle model. This experiment also indicated that metabolism of the test chemicals decreases their inhibition potency in inhibiting T_4_ and T_3_ production since preincubation with liver organoids decreased the inhibition. The latter is an important consideration when incorporating a metabolizing tissue into any bioassay since the bioactivity of the test chemicals are often impacted.

#### 4.2.7 Expression and variability of T_4_ and T_3_ values

There are several ways to calculate the effect of a test chemical on T_4_ and T_3_ levels: nM, pmoles/million cells, % initial levels, % of solvent control values and the T_4_:T_3_ ratio. Each of these have advantages, for example, the use of nM units enables a direct comparison of concentrations in the Chip3 with *in vivo* plasma concentrations (which were comparable when freshly isolated thyroid follicles were used but 10-fold lower when thyrocyte-derived thyroid follicles were used). However, if values in solvent controls decrease over time, the impact of the test chemicals is best visualized as a % of the initial levels or of solvent control values at each timepoint. The T_4_:T_3_ ratio has the advantage that it compares the relative concentrations of T_4_ and T_3_ in the same sample which is independent of the number of functional thyroid follicles in the circuit and therefore provides a more robust reflection of the impact of the test chemical. The T_4_:T_3_ ratio is also used as a clinical marker for patients with thyrotoxicosis (Mortoglou and Candiloros, 2004; Narkar et al., 2021), making this an especially relevant marker for the Chip3 experiments. Regardless of the unit, the values for hormone levels were more variable than measurements of test chemical concentrations in the same medium samples. For example, the mean %CVs for T_4_ and T_3_ expressed as nM across all circuits (solvent control and test chemical samples) and timepoints were 47% and 45%, respectively, whereas the mean %CV for the measurement of the concentrations of test chemicals was only 12%. The variability in hormone production across circuits is likely to be due to the different number of functional thyroid follicles present in the beads, indicating that the culture method needs further optimization to standardize the follicle size and thus production of hormones.

#### 4.2.8 Test chemical distribution

It is important to confirm the biokinetics of test chemicals in any *in vitro* assay. A lack of bioactivity could be due to a lack of intracellular exposure or, indeed of the tissue itself. In the final Chip3 experiment, the distributions of the test chemicals were measured in all three organoids, the medium and residual chip wash at the end of the 5-day incubation. Importantly, in each dosing scenario, the thyroid contained 15%–20% of the total chemical present, indicating that this target organoid was exposed to genistein and daidzein. Interestingly, very little of the test chemicals were present in the liver spheroids, apart from after repeated systemic application of 58 µM daidzein due to some precipitation of daidzein on liver spheroids.

Only 2% of the topical “safe dose” of daidzein remained on the skin surface 24 h after its last application, indicating that most had entered the skin and the systemic circulation. Approximately 20% of the LOEL dose (which was 10-fold higher than the “safe dose”) remained on the skin surface, suggesting that absorption of this dose was not complete. Analysis of the metabolites showed a similar distribution, with the exception that no metabolites were recovered in the skin washes or chip washes (data not shown).

### 4.3 Use of the Chip3 for safety assessment

Having established suitable conditions for the co-culture of thyroid follicles with Phenion FT models and liver spheroids in the Chip3, this model was then used in a hypothetical safety assessment to determine the highest concentration of daidzein that can be used in a body lotion without effects on thyroid hormones. In reality, the safety assessment of this chemical and the point of departure is based on its estrogenic effects (Scientific Committee on Consumer Safety, 2022); however, a tissue model for the estrogen receptor is currently unavailable, making it impossible to base the current evaluation on this endpoint. Since endocrine effects are important not just the safety assessment of cosmetics but of a wide range of chemicals (pesticides, fungicides, industrial chemicals, plasticizers, non-ylphenols, metals, pharmaceutical agents and phytoestrogen (Yilmaz et al., 2020), further investments into expanding this model, especially for dermal exposure, would be highly beneficial.

The first Chip3 experiment in which genistein and daidzein were tested indicated that the doses selected were not “no effect” doses. For this reason, a second experiment was performed using 2.35 µg/cm^2^ as the LOEL dose for daidzein and a 10-fold lower dose as a potential “safe dose”. Due to the novelty of the test system, the use of the standard conversion of 3-fold from an *in vivo* data-based LOEL to a NOEL [according to Yang et al. (2017)] was considered insufficient to provide confidence in the NOEL with respect to the resulting hormone inhibition; therefore, the overall safety factor was increased to 10. This meant that the difference between the LOEL and the “safe dose” contained a further safety factor of 3.3 to increase confidence that the estimated NOEL concentration lacked bioactivity (i.e., did not affect T_4_ or T_3_). This 10-fold safety factor was supported by the data i.e., that repeated application of the LOEL dose altered the T_4_ and T_3_ concentrations, whereas the “safe dose” did not. It was thus concluded that a topical dose of 0.235 µg/cm^2^ could be considered as safe with respect to thyroid effects. The corresponding circuit concentrations of daidzein was 0.30 µM.

The dose of 0.235 µg/cm^2^ equates to a safe concentration in a body lotion of 0.047% daidzein applied to the whole body (according to a surface area of 15,670 cm^2^ exposed to 0.5 mg/cm^2^ (Scientific Committee on Consumer Safety, 2022)). Importantly, this value is only marginally higher than the concentration of 0.02% daidzein considered safe by the SCCS for use in cosmetic products (Scientific Committee on Consumer Safety, 2022). Of note, the safe concentration of daidzein derived in the SCCS opinion is not directly comparable to the result here since it builds on the most sensitive parameter i.e., the potential estrogen disrupting activity. However, it does indicate that the value derived from the Chip3 model was realistic in that it predicted a concentration which was in the same order of magnitude as that in the SCCS opinion. A higher safe concentration of daidzein than genistein may be expected based on the difference in their bioactivity potencies in this study (e.g., T_4_ inhibition IC_50_ values for genistein and daidzein were 54 µM and 152 μM, respectively), which also correlates with the concentrations of 0.02% daidzein and 0.007% genistein considered by the SCCS to be safe.

Further studies are required to challenge the predictive capacity of the Chip3 model using additional chemicals, and to evaluate the protective nature of the factors chosen to determine safe topical doses. Combinations of benchmark dose-based experiments to identify reasonable points of departure for risk assessment may also help to increase confidence, as well as show the benefits of incorporating toxicokinetic parameters in a combined system.

## 5 Conclusion

In conclusion, we have developed a skin-liver-thyroid Chip3 co-culture model to evaluate the effects of topically applied chemicals on thyroid hormone production. This skin-liver-thyroid Chip3 model is closer to the *in vivo* situation compared to more traditional *in vitro* static monoculture methods. The optimization steps highlighted several aspects that should be considered in future experimental designs for these types of studies, including medium composition, standardization of thyrocyte-derived thyroid follicle sizes, methods for dose-setting and use of appropriate solvents. In a hypothetical safety assessment, the resulting model provided a realistic estimate of the concentration of daidzein in a body lotion formulation which does not alter T_3_ and T_4_ levels. While the model still requires additional optimization, this study demonstrated its applicability in assessing the potential thyroid-related endocrine disrupting effects of repeated topically exposed chemicals.

## Data Availability

The original contributions presented in the study are included in the article/Supplementary Material, further inquiries can be directed to the corresponding author.

## Abbreviations

FCS, fetal calf serum; FT, Full Thickness; LDH, lactate dehydrogenase; LOEC, lowest observed effect concentration; LOEL, lowest observed effect level; MMI, methimazole; MPS, microphysiological systems; NOEC, no observed effect concentration; NOEL, no observed effect level; PBPK, physiologically based pharmacokinetic; PoD, point of departure; TSH, thyroid stimulating hormone; T_4_, thyroxine; T_3_, 3,3′,5-triiodothyronine; TEER, transepithelial electrical resistance.
