# Supplementary material for: Development of a microphysiological skin-liver-thyroid Chip3 model and its application to evaluate the effects on thyroid hormones of topically applied cosmetic ingredients under consumer-relevant conditions
Source: Front Pharmacol. 2023 Feb 8;14:1076254. doi: 10.3389/fphar.2023.1076254 (PMC9946709; doi:10.3389/fphar.2023.1076254)
Supplement: Supplementary file 1 [file DataSheet1.pdf]

Supplementary Material

1 Materials

Cells, tissue			
**Product**	**Article number**	**Supplier**	**Lot**
HepaRG	HPR116	Biopredic International, Rennes, France	HPR116239-TA08
HepaRG	HPR116080	Biopredic International, Rennes, France	HPR116308-TA08
Human Hepatic Stellate Cells	5300	ScienCell, Carlsbad, CA, USA	
Primary thyroid tissue	Not applicable	Provio, Germany	13202007TG112
Phenion FT skin modell in Millicell 24-w standing insert (42x)	FT INSERT24-1	Phenion, , Düsseldorf, Germany	
**Media, buffers and supplements for cell culture**			
**Product**	**Article number**	**Supplier**	**Lot**
Amphotericin	30-003-CF	Corning, Glendale, USA	27118006
BSA 30%	A8327-50ML	Sigma, Taufkirchen, Germany	
bTSH	T8931	Calbiochem	3099973
Charcoal stripped FBS	F6765	Sigma, Taufkirchen, Germany	19M018
Charcoal stripped FBS	A33821-01	ThermoFisher Scientific, Germany	
Collagenase	S1745403	Nordmark Biochemicals, Uetersen, Germany	
Cultrex® Stem Cell Qualified Reduced Growth Factor Basement Membrane Extract	3434-005-02	R&D Systems, Minneapolis, USA	1636972
Daidzein	D7802	Sigma, Taufkirchen, Germany	128M4149V
Dispase II	D4693	Sigma, Taufkirchen, Germany	
DMSO	23500260	VWR, Darmstadt, Germany	
FCS	35-079-CV	Corning, Glendale, USA	35079002
FCS	10	ScienCell, Carlsbad, CA, USA	27001
Genistein	AG-CN2-0427-M250	AdipoGen, Hamburg, Germany	A000495
Gentamycin sulfat	30-005-CR	Corning, Glendale, USA	19619009
Glucose 45%	25-037-CIR	Corning, Glendale, USA	
Glutagro Supplement	25-015-CI	Corning, Glendale, USA	5020010
Hydrocortisone 21-hemisuccinate	H4881-1G	Sigma, Taufkirchen, Germany	SLBP7869V
Insulin	P07-04300	PAN Biotech, Aidenbach, Germany	6440120
L-Glutathione reduced	G6013-5G	Sigma, Taufkirchen, Germany	SLCH4329
Matrigel® Growth Factor Reduced (GFR) Basement Membrane Matrix, LDEV-free	354230	Corning, Glendale, USA	
Methimazole	301507	Sigma, Taufkirchen, Germany	WXBC9951V
Nutrient Mixture F-12 Ham	F6636-1L	Sigma, Taufkirchen, Germany	SLCC7339
P/S Antibiotic solution	503	ScienCell, Carlsbad, CA, USA	
Recombinant human Growth Hormone (E.coli derived)	1067-GH/CF	R&D Systems, Minneapolis, USA	FTO0319101

SILAC Advanced DMEM/F12 Flex w/o phenol red	A24943-01	ThermoFisher Scientific, Germany	
Sodium Bicarbonate	S5761-500g	Sigma, Taufkirchen, Germany	BCCB6896
Sodium Selenite	A2776	AppliChem, Darmstadt, Germany	1Y000571
SteC GS	5352	ScienCell, Carlsbad, CA, USA	27001, 30305
SteCM	5301	ScienCell, Carlsbad, CA, USA	27169, 30187
Trypsin/EDTA	25-053-CI	Corning, Glendale, USA	
William's E, phenol red-free	P04-29510	PAN Biotech, Aidenbach, Germany	

2 Calculation of the systemic doses of genistein and daidzein in the Chip3

To ensure systemic doses approximating the LOECs in the Chip3, the metabolism of the test chemicals by liver organoids (i.e. first-pass metabolism in the Chip) was taken into account. This was achieved by adjusting the nominal doses according to the slope of the correlation of the nominal concentration and the concentration remaining after 24 h incubation with liver spheroids.

Table 1. Comparison of the nominal concentrations of genistein added to liver spheroids with measured concentrations after four consecutive days of incubation (for 24 h).

Nominal concentration (nM)	Concentration (nM) in liver spheroid incubations after 24 h				Mean remaining concentration (nM)	
	1st dose	2nd dose	3rd dose	4th dose	Mean	SD
20	4	4	7	12	7	3.6
60	106	46	39	37	57	32.7
200	122	118	122	156	130	17.7
600	406	411	375	454	411	32.6
2000	1185	1230	1419	1435	1317	128.2
6000	3051	3407	2742	3329	3132	301.9
20000	10957	12813	12766	13791	12582	1181.8
60000	35752	49375	51783	51294	47051	7603.8

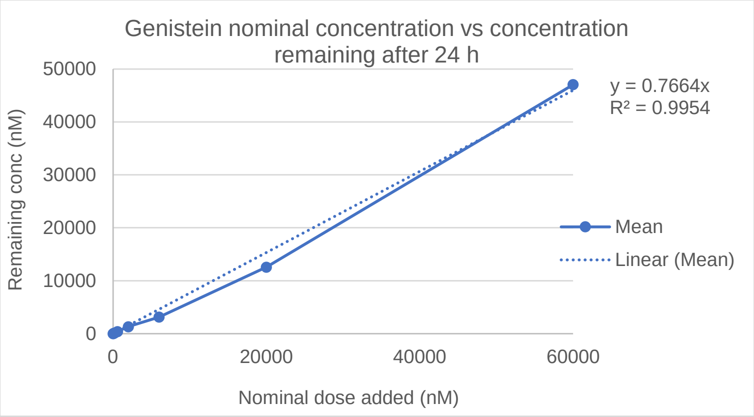

0.7664	= slope
19	= Target conc in Chip3 (µM)
25	= Adjusted dose (µM)

Table 2. Comparison of the nominal concentrations of daidzein added to liver spheroids with measured concentrations after four consecutive days of incubation (for 24 h).

Nominal concentration (nM)	Concentration (nM) in liver spheroid incubations after 24 h				Mean remaining concentration (nM)	
	1st dose	2nd dose	3rd dose	4th dose	Mean	SD
20	6	9	8	8	8	1.2
60	21	17	21	20	20	1.8
200	52	57	78	58	61	11.6
600	200	251	171	226	212	34.5
2000	827	672	643	746	722	82.6
6000	1663	2493	1768	1827	1938	376.2
20000	8199	8482	9214	9708	8901	687.2
60000	17716	29096	32624	37534	29243	8427.7

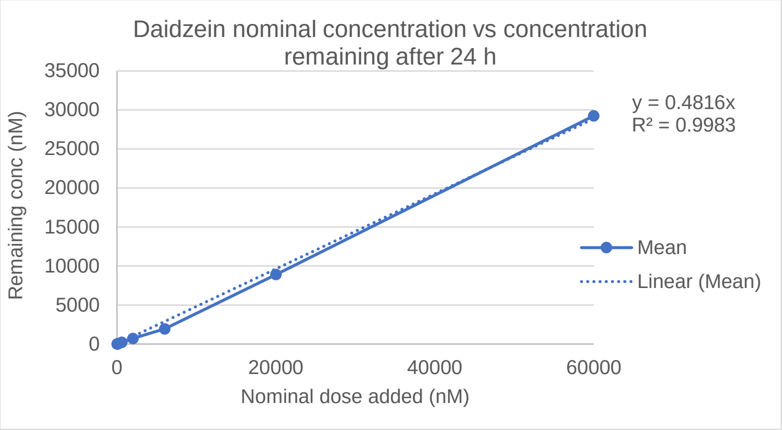

0.4816	= slope
28	= Target conc in Chip3 (µM)
58	= Adjusted dose (µM)

The adjustment factors for genistein and daidzein were 1.30 and 2.1, respectively, resulting in doses of 25 µM genistein and 58 µM daidzein being applied.

3 Supplementary Data Targeted testing of endocrine activity using CALUX assays

Genistein and daidzein were tested in a panel of CALUX® transactivation assays to investigate potential MoAs for reproductive toxicity [1-3].

Methods

Determination of cytotoxicity

Before analysis on the various bioassays, the cytotoxicity of genistein and daidzein was assessed using the U2-OS based CALUX cytotox bioassay. The cytotox CALUX cells constitutively express luciferase. Exposure of the cytotox CALUX cells to chemicals causing cytotoxicity results in a reduction of luminescence. Chemical concentrations causing >20% reduction of luminescence are considered cytotoxic.

hTPO inhibition assay

hTPO was derived from Nthy-ori 3-1 cells. Cell lysate containing hTPO in Glycine-NaOH buffer (pH 9.0) was incubated for 30 min at 37°C in the presence of serial dilutions of the chemicals in DMSO (1% chemical stock in incubation mixture). The incubation mixture was transferred to 96-well microtiter plates after which luminol (34.8 µM) and H2O2 (1.7 mM) were added. Luminescence was measured on a Berthold luminometer.

TTR-binding assay

Serial dilutions of the chemicals were incubated in Tris-buffer (pH 8.0) overnight at 4oC in the presence of TTR (0.058 µM) and a fixed concentration of T4 (0.052 µM) (3.2% chemical stock in incubation mixture). After incubation, TTR-bound and free T4 were separated on a Bio-Gel P-6DG column. The eluate was added to assay medium after which TRβ CALUX cells were exposed for 24 h. For this TRβ CALUX exposure, serum-free assay medium was used.

Results

The cytotoxicity of genistein and daidzein was tested to ensure that the EATS assay outcomes were not impacted by cytotoxic effects. Neither chemical was cytotoxic up to the highest concentration of 10 mM, in the absence or presence of rat liver S9. The rat liver S9 was incubated with cofactors that mediate phase 1 pathways (i.e. an NADPH and an NADPH-regenerating system); therefore, since genistein and daidzein are only conjugated via phase 2 pathways, the cytotoxicity was not expected to be altered by the inclusion of S9.

Genistein and daidzein did not activate or antagonize the thyroid receptor, either in the absence or the presence of rat liver S9. Both genistein and daidzein inhibited TPO activity, with a PoD (LOEC) of 1.9×10-5 ± 8.9×10-6 M for genistein and 2.8×10-5 ± 1.3×10-5 M for daidzein. Genistein inhibited binding of T4 to TTR at a concentration of 2.1×10-7 ± 6.7×10-8 (LOEC), and daidzein was slightly less potent, with a LOEC of 1.0×10-6 ± 0.

References

1. Collet B, Simon E, van der Linden S, el Abdellaoui N, Naderman M, Man H-y, et al. Evaluation of a panel of in vitro methods for assessing thyroid receptor β and transthyretin transporter disrupting activities. Reproductive Toxicology. 2019.

2. van der Burg B, Wedebye EB, Dietrich DR, Jaworska J, Mangelsdorf I, Paune E, et al. The ChemScreen project to design a pragmatic alternative approach to predict reproductive toxicity of chemicals. Reprod Toxicol. 2015;55:114-23.

3. van Vugt-Lussenburg BMA, van der Lee RB, Man HY, Middelhof I, Brouwer A, Besselink H, et al. Incorporation of metabolic enzymes to improve predictivity of reporter gene assay results for estrogenic and anti-androgenic activity. Reprod Toxicol. 2018;75:40-8.

4 Supplementary Figures

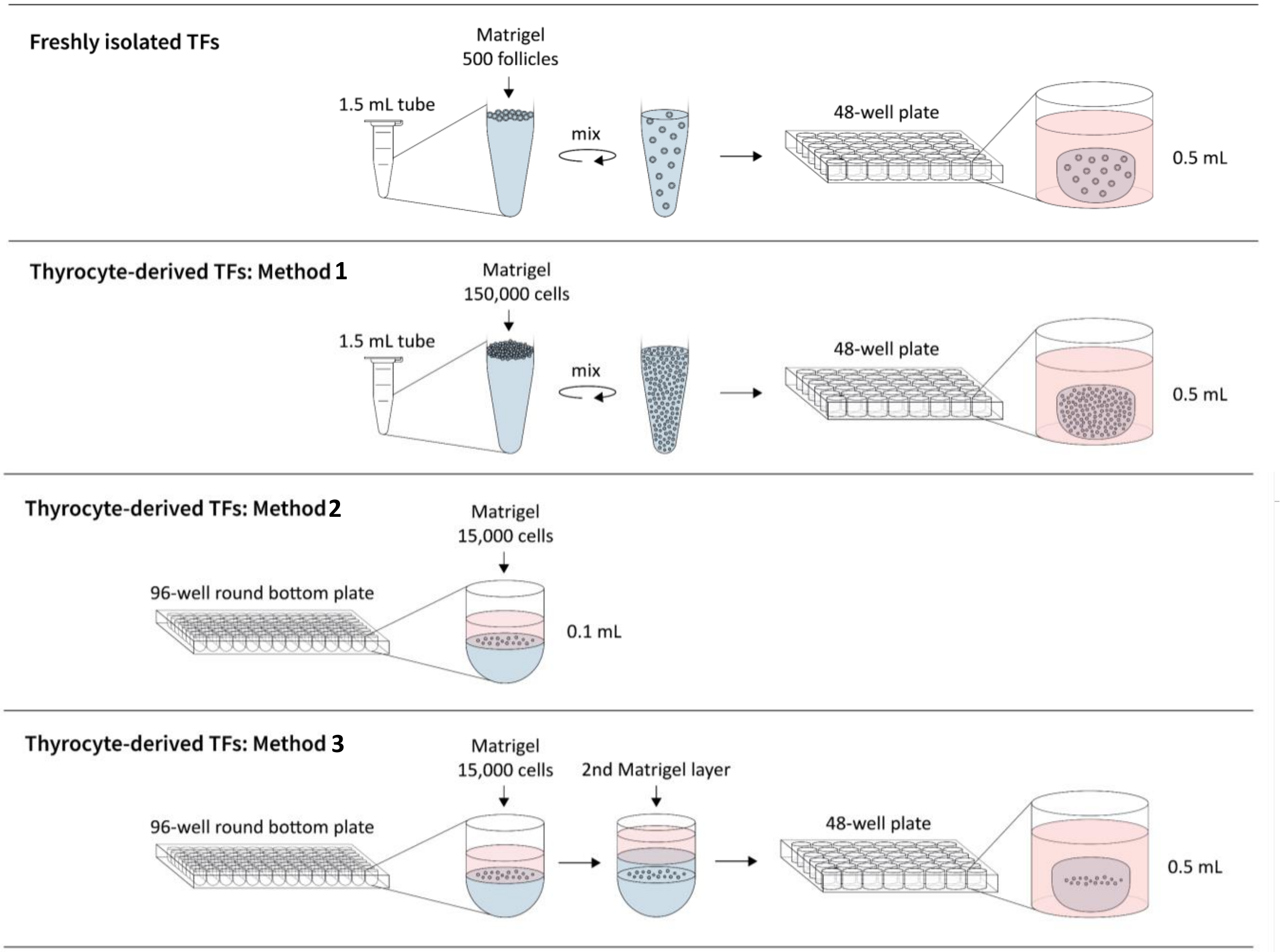

Supplemental Figure 1. An overview of the culture methods for freshly isolated and thyrocyte-derived thyroid follicles.

frontiers

Experiment desscription	Culture type	Thyrocyte method	Cell No./model	No. thyroid models/incubation	Culture volume	Thyrocyte donor(s)	Day of culture (thyroid model)																
							D0	D1	D2	D3	D4	D5	D6	D7	D8	D9	D10	D11	D12	D13	D14	D15	D16
Media test	Static	NA	1,000 or 500 follices	1	0.5 mL	NA - primary thyroid follicles		x	x			x		x									
Media test	Static	2	15,000 cells	1	0.1 mL	Donor 1		x			x		x		x		x<br>MMI x1		x				
Pilot - Confirmation of hormone stimulation by TSH in Chip3	Chip3	1	150,000 cells	1	1 mL	Donor 1	Static	x<br>Static	Static	Static	x<br>Static	Static	x<br>Static	Static	x<br>Chip equil		x		x<br>MMI x1		x		
Thyrocyte culture format comparison	Static	1, 2 & 3	1) 150,000 cells<br>2) 15,000 cells<br>3) 15,000 cells	1	1. 0.1 mL<br>2. 0.5 mL<br>3. 0.5 mL	Donor 1, 2 and 3			x			x		x			x<br>MMI x1<br>(D1 only)		x				
T3/T4 dose response curve for genistein & daidzein	Static	3	15,000 cells	2	0.5 mL	Donor 1 and 4		x			x			x<br>TC/MMI	x<br>TC/MMI	x<br>TC/MMI	x<br>TC/MMI						
Impact of application route on T3/T4 in Chip3 - 1st experiment	Chip3	3	15,000 cells	4	1 mL	Donor 2	Static	Static	Static	x<br>Static	x<br>Overlay	Chip equilib	x<br>TC/MMI	x<br>TC/MMI	x<br>TC/MMI	x<br>TC/MMI							
Impact of application route on T3/T4 in Chip3 - 2nd experiment	Chip3	3	15,000 cells	4	1 mL	Donor 4 and 6	Static	Static	Static	x<br>Static	x<br>Overlay	Chip equilib	x<br>TC/MMI	x<br>TC/MMI	x<br>TC/MMI	x<br>TC/MMI	x<br>TC/MMI						

Thyrocyte culture methods
1 According to Deisenroth et al.
2 Modification of Deisenroth, Matrigel overlay
3 Matrigel-entrapment

x = medium change
TC = test chemical
MMI = Methimazole
Chip equil = equilibration of organoids in the chip circuit
Overlay = addition of 0.1 mL Matrigel to thyroid cultures
Static = static culture preincubation

Blue = medium + FCS + TSH
Orange = medium + BSA ± TSH
Purple = Chip3 medium + BSA ± TSH
Green = Chip3 medium + BSA + TSH

Supplementary Figure 2. An overview of the designs of the static and Chip3 experiments.

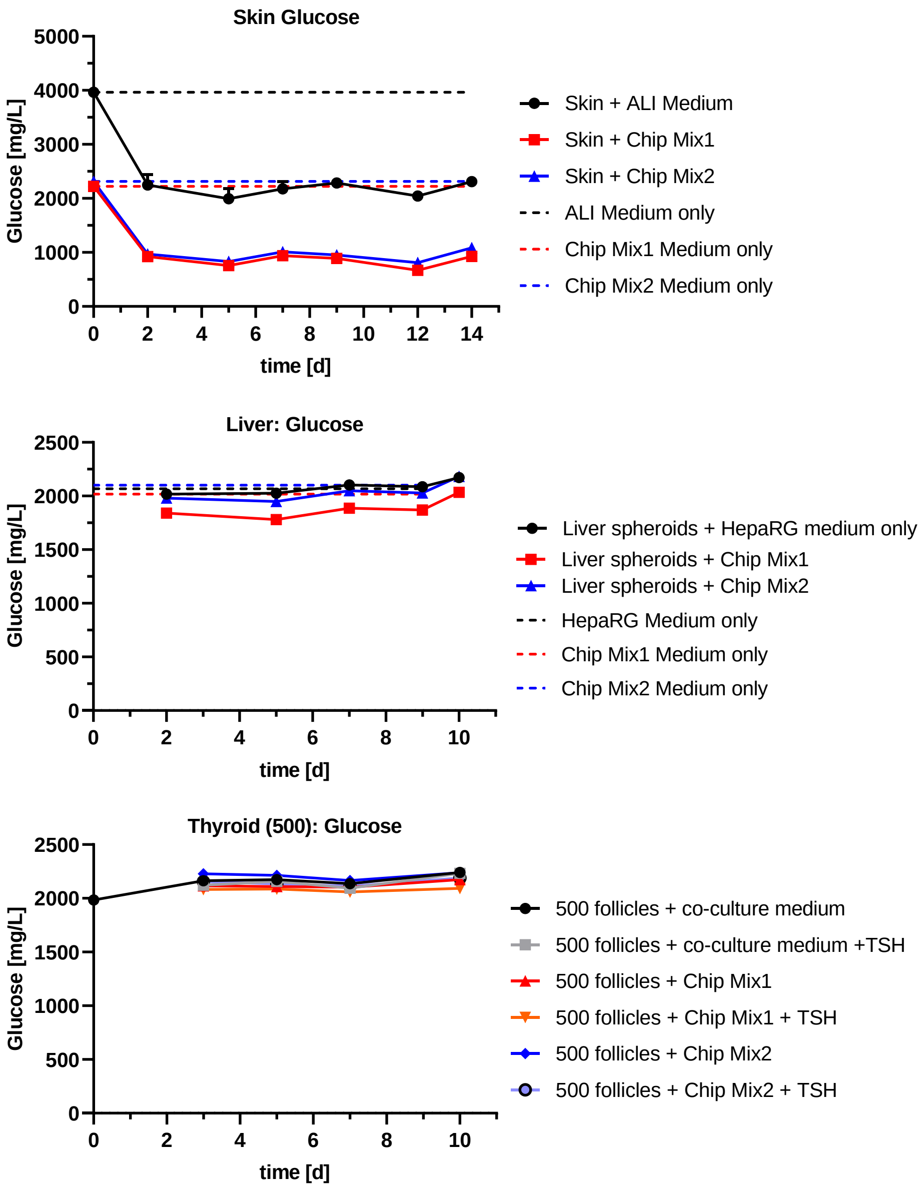

Supplementary Figure 3A. Viability according to glucose concentrations of freshly isolated human thyroid follicles (500 per incubation), Phenion FT models and liver spheroids in different media. Values are mean ± SD, n= 6 samples were incubated for each organoid. Values denoted with dotted lines indicate the concentrations measured in the corresponding medium in the absence of organoids. The media tested were 100% ALI medium (for skin), 100% HepaRG medium (for liver spheroids) and 100% liver-thyroid co-culture medium (for thyroid follicles) and a mix of liver-thyroid co-culture medium and skin ALI medium (in a ratio of 50:50 (Mix 1) or 70:30 (Mix 2)), all thyroid follicle incubations were with or without the addition of 1 mU/mL TSH.

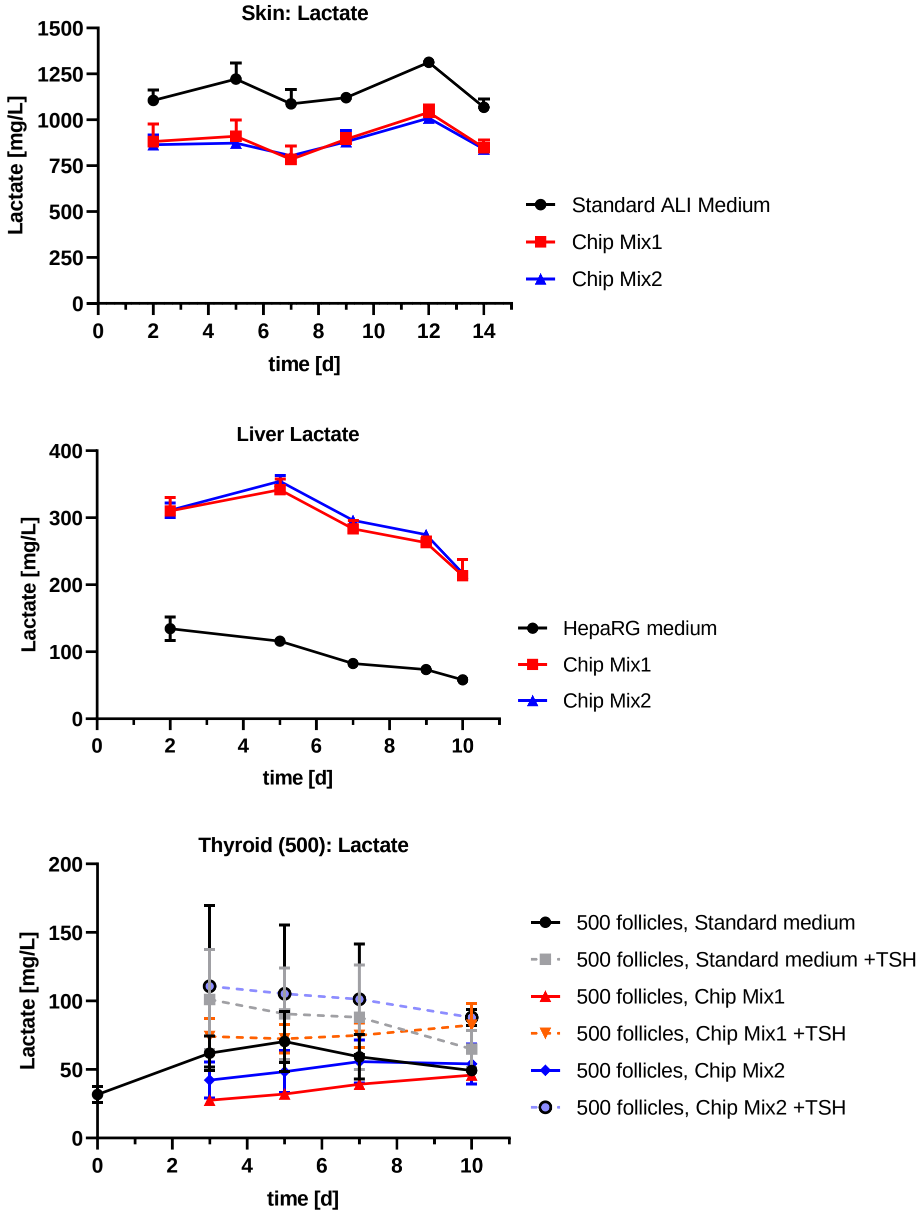

Supplementary Figure 3B. Viability according to lactate concentrations of in incubations with freshly isolated human thyroid follicles (500 per incubation), Phenion FT models and liver spheroids in different media. Values are mean ± SD, n= 6 samples were incubated for each organoid. The media tested were 100% ALI medium (for skin), 100% HepaRG medium (for liver spheroids) and 100% liver-thyroid co-culture medium (for thyroid follicles) and a mix of liver-thyroid co-culture medium and skin ALI medium (in a ratio of 50:50 (Mix 1) or 70:30 (Mix 2)), all thyroid dfollicle incubations were with or without the addition of 1 mU/mL TSH.

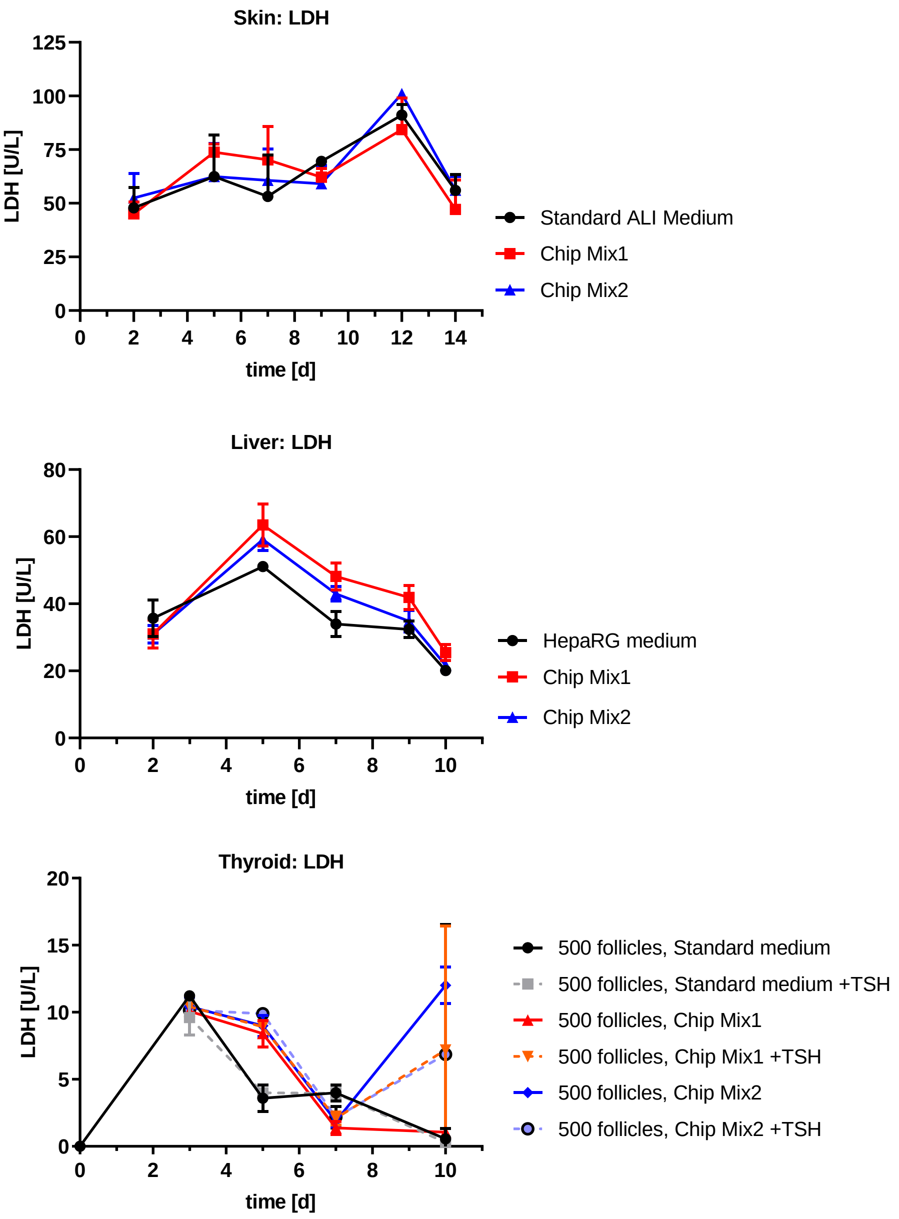

Supplementary Figure 3C. Viability according to LDH release in incubations of freshly isolated human thyroid follicles (500 per incubation), Phenion FT models and liver spheroids in different media. Values are mean ± SD, n= 6 samples were incubated for each organoid. The media tested were 100% ALI medium (for skin), 100% HepaRG medium (for liver spheroids) and 100% liver-thyroid co-culture medium (for thyroid follicles) and a mix of liver-thyroid co-culture medium and skin ALI medium (in a ratio of 50:50 (Mix 1) or 70:30 (Mix 2)), all thyroid follicle incubations were with or without the addition of 1 mU/mL TSH.

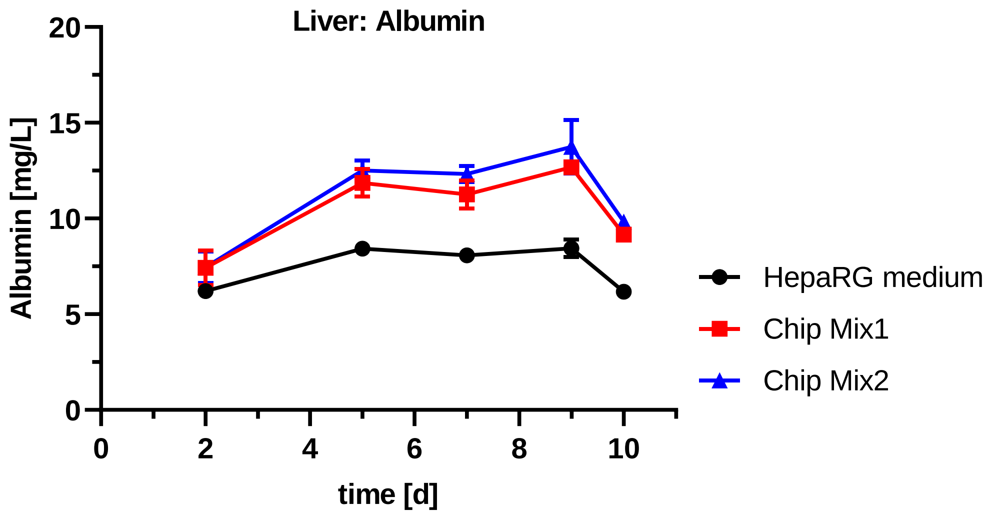

Supplementary Figure 3D. Viability of liver spheroids in different media according to albumin production. Values are mean ± SD, n= 6 samples were incubated. The media tested were 100% HepaRG medium and a mix of liver-thyroid co-culture medium and skin ALI medium (in a ratio of 50:50 (Mix 1) or 70:30 (Mix 2)).

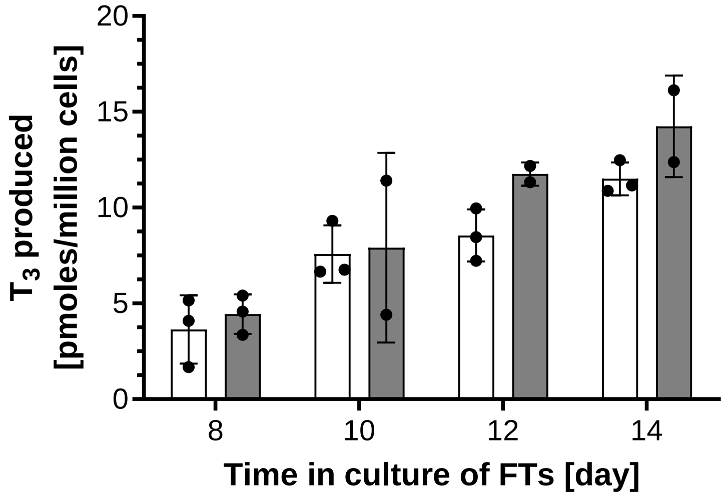

Supplementary Figure 4. Basal and 1 mU/ml TSH-stimulated hormone production in static (Method 2 thyroid follicles) incubations from Donor 1 over time. Basal (white bars) and TSH-stimulated (grey bars) hormone production is expressed as a mean + SEM of pmoles/million cells, n= 3 (static).

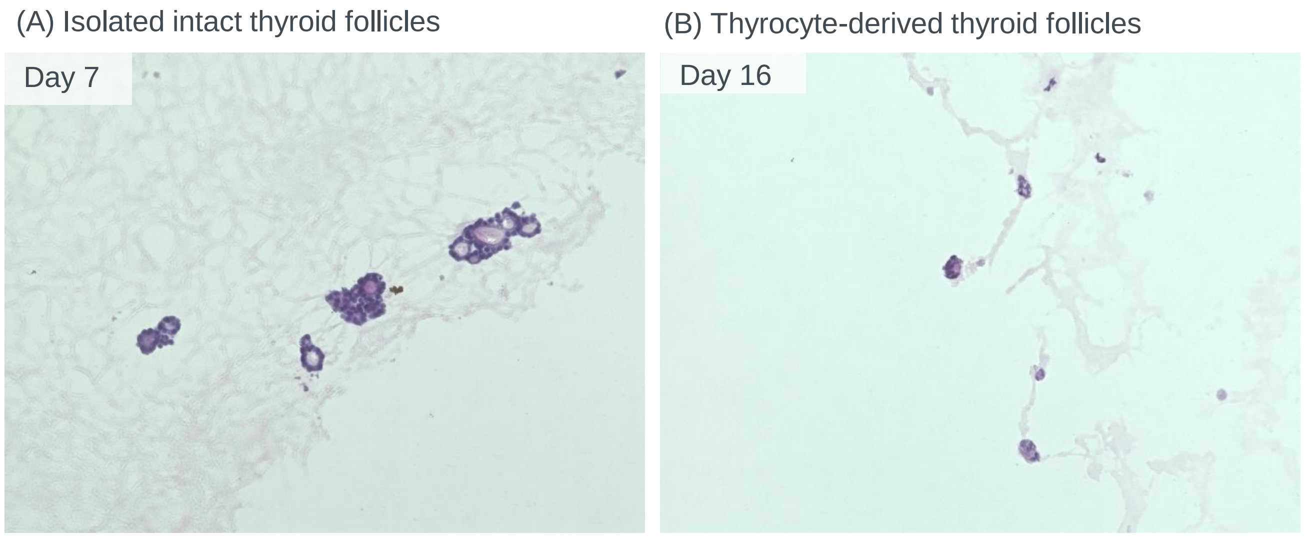

Supplementary Figure 5. Morphology of freshly isolated intact thyroid follicles (A) and human thyrocyte-derived thyroid follicles using Method 3 (B). Images of H&E staining are at a x20 magnification.

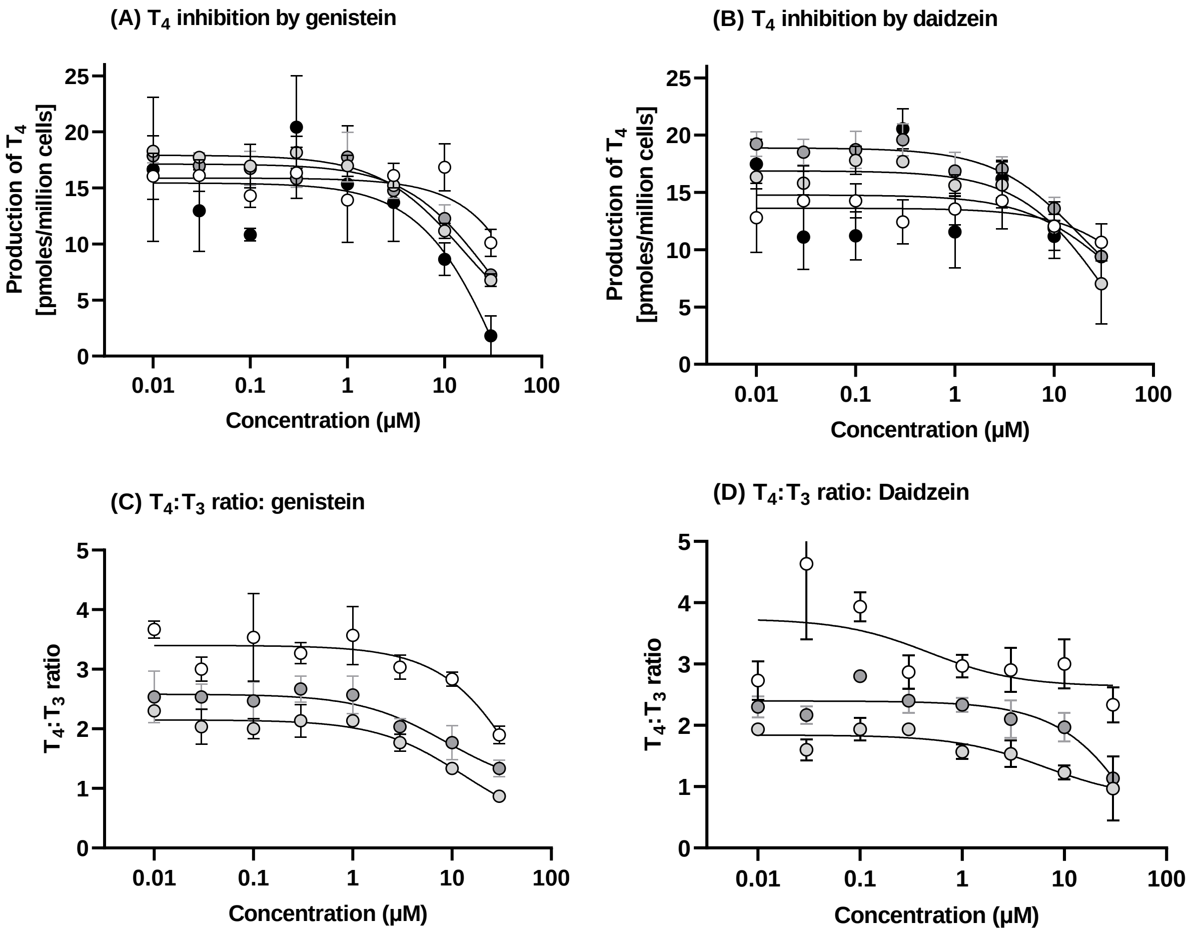

Supplementary Figure 6. Inhibition of T_4 production by thyroid follicles (A and B) and T_4:T_3 ratios (C and D) in static cultures (Method 2) treated with repeated daily and increasing concentrations of genistein (A and C) and daidzein (B and D). Values are expressed as a mean ± SEM of pmoles/million cells, n= 3. Symbols are as follows: ○ = Day 1, ○ = Day 2, ● = Day 3, ● = Day 4

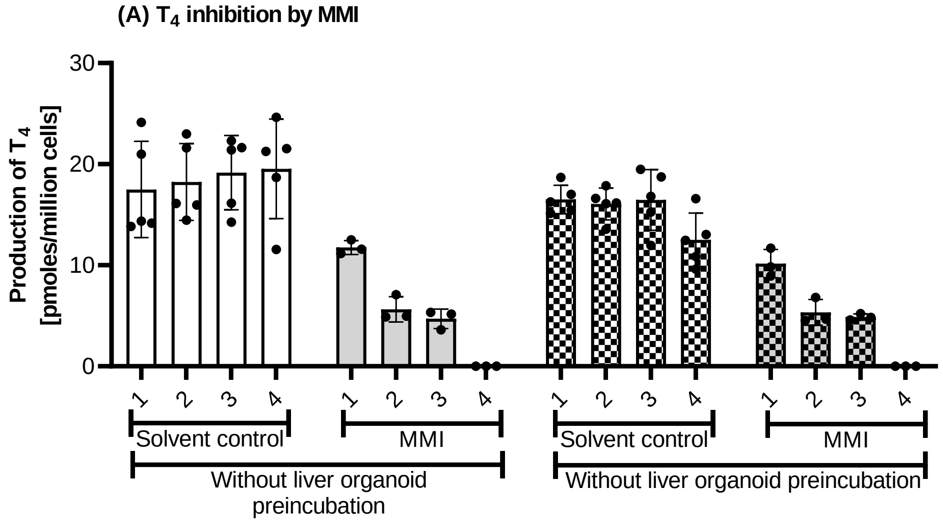

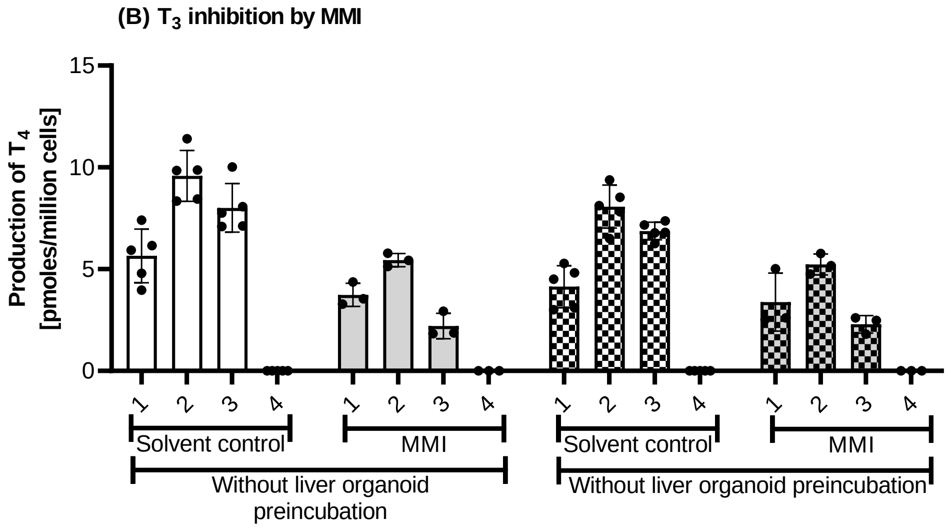

Supplementary Figure 7. TSH (1 mU/mL) stimulated T_4 (A) and T_3 (B) production by thyroid follicles in static culture (Method 3) treated with repeated daily applications of the positive control compound, MMI (10 µM) or the solvent control DMSO. Values are expressed as a mean + SEM of pmoles/million cells, n= 3.

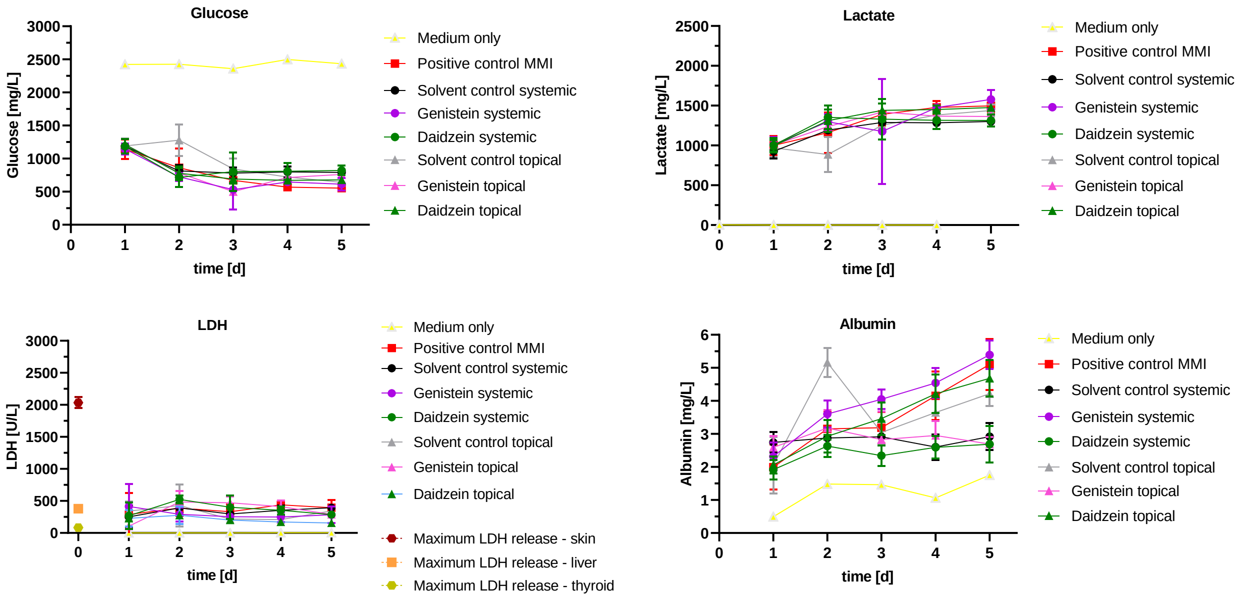

Supplementary Figure 8. Viability of organoids in the first main Chip3 experiment according to glucose concentrations (A), lactate concentrations (B), LDH release (C) and albumin production by liver spheroids (D). Values are mean ± SD, n=5 Chip3 circuits. For the systemic applications, the solvent control for MMI was 0.1% DMSO and the solvent control for genistein and daidzein was 2% ethanol. The maximum amount of LDH that can be released by the organoids are denoted by a symbol at time zero. The concentrations of glucose and albumin in medium only (in the absence of organoids) are also denoted in the figures.

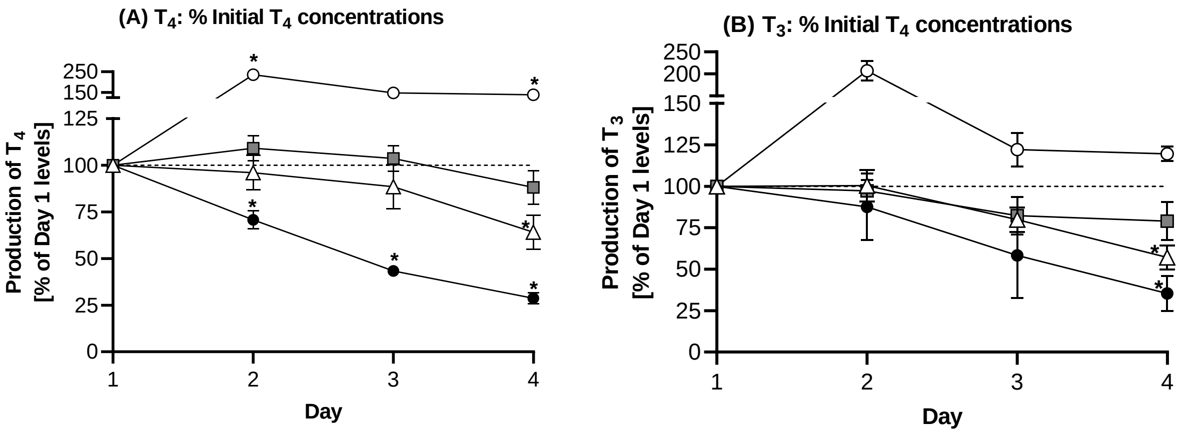

Supplementary Figure 9. T_4 (A), T_3 (B) levels in the Chip3 expressed as the initial levels over 4 days after repeated daily topical applications of a lotion formulation containing 1.55 µg/cm^2 genistein (white triangles) or 2.35 µg/cm^2 daidzein (grey squares) or the lotion formulation alone (white circles). The positive control was 10 µM MMI (black circles), applied systemically daily at each medium change. Values are expressed as a mean ± SEM of the percentage of the T_4 or T_3 concentration on Day 2, 3 or 4 compared to levels on Day 1, n= 5 circuits. Values which are statistically significantly ($p<0.05$, 2-way ANOVA) different from Day 1 are denoted with an asterisk.

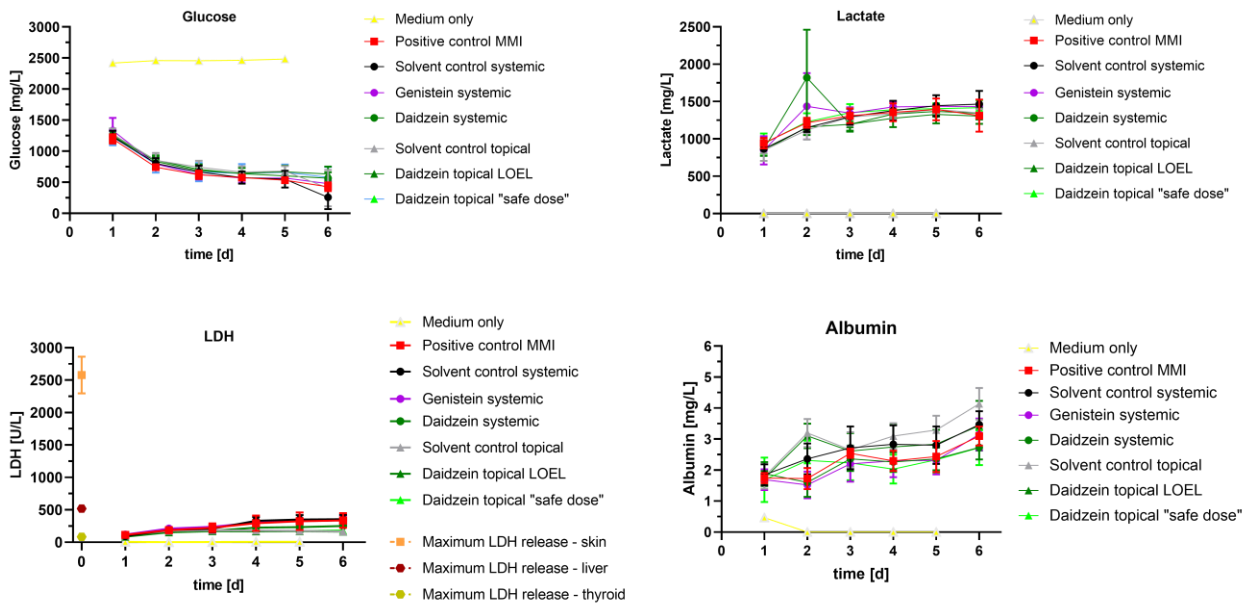

Supplementary Figure 10. Viability of organoids in the second main Chip3 experiment according to glucose concentrations (A), lactate concentrations (B), LDH release (C) and albumin production by liver spheroids (D). Values are mean ± SD, n=5 Chip3 circuits. For the systemic applications, the solvent control for MMI was 0.1% DMSO and the solvent control for genistein and daidzein was 0.1% DMSO. The maximum amount of LDH that can be released by the organoids are denoted by a symbol at time zero. The concentrations of glucose and albumin in medium only (in the absence of organoids) are also denoted in the figures.

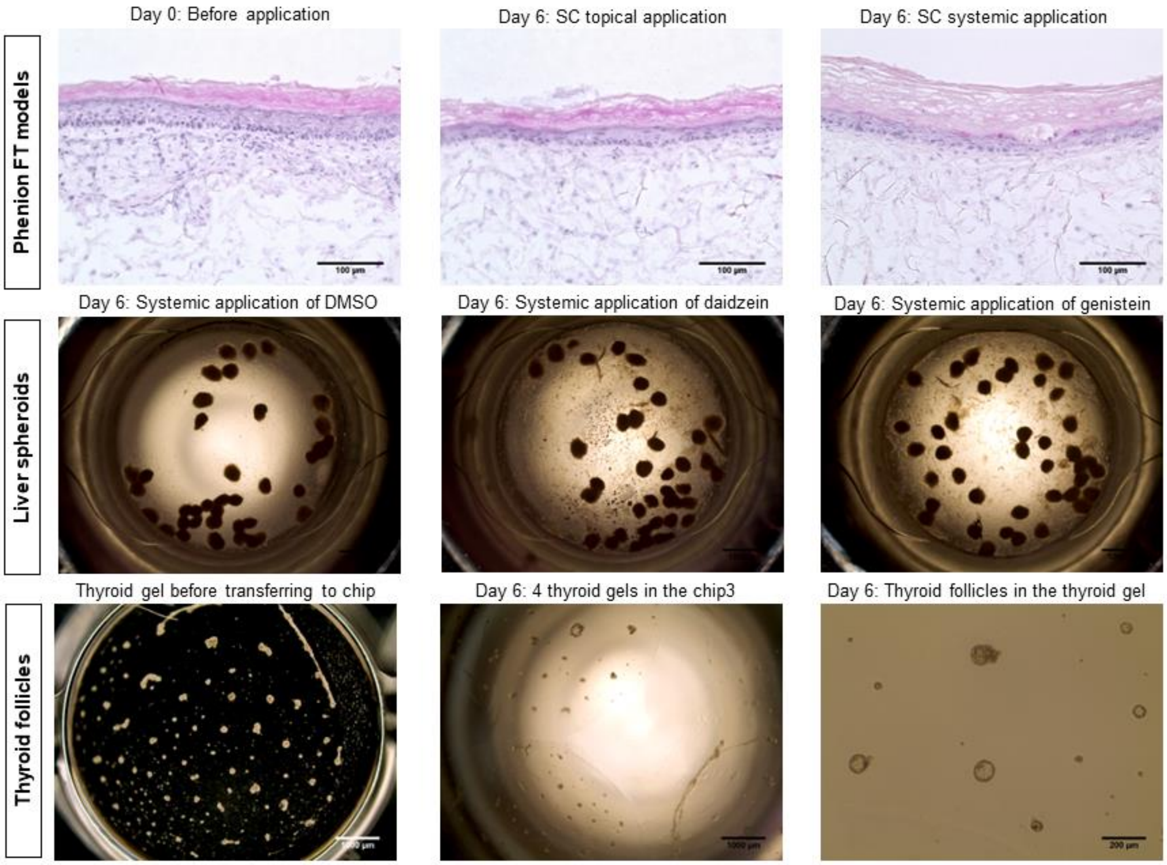

Supplementary Figure 11. Morphology of skin, liver and thyroid models in the Chip3 model after different application scenarios. Images were selected to show that (1) the Phenion FT skin models (captured from the first Chip3 experiment) retained their structure over time even after application of the lotion formulation; (2) liver spheroids exhibited some outgrowth of cells (indicating the medium needs optimization) and daidzein exhibited marked precipitation, evident as black particles and (3) thyrocytes formed dense aggregates after 4 days of static culture, and the typical spherical morphology of thyroid follicles with lumen was observed after a few days in the chip. Images of each organoid were captured from all circuits – 3-5 circuits per treatment.
